# Highly multiplexed 2-dimensional imaging mass cytometry analysis of HBV-infected liver

**DOI:** 10.1172/jci.insight.146883

**Published:** 2021-04-08

**Authors:** Daniel Traum, Yue J. Wang, Kathleen B. Schwarz, Jonathan Schug, David K.H. Wong, Harry L.A. Janssen, Norah A. Terrault, Mandana Khalili, Abdus S. Wahed, Karen F. Murray, Phillip Rosenthal, Simon C. Ling, Norberto Rodriguez-Baez, Richard K. Sterling, Daryl T.Y. Lau, Timothy M. Block, Michael D. Feldman, Elizabeth E. Furth, William M. Lee, David E. Kleiner, Anna S. Lok, Klaus H. Kaestner, Kyong-Mi Chang

**Affiliations:** 1 Department of Medicine, University of Pennsylvania Perelman School of Medicine, Philadelphia, Pennsylvania, USA.; 2 Medical Research, The Corporal Michael J. Crescenz VA Medical Center, Philadelphia, Pennsylvania, USA.; 3 Biomedical Sciences, College of Medicine, Florida State University, Tallahasee, Florida, USA.; 4 Department of Pediatrics, UCSD, San Diego, California, USA.; 5 Department of Genetics and Institute for Diabetes, Obesity, and Metabolism, University of Pennsylvania Perelman School of Medicine, Philadelphia, Pennsylvania, USA.; 6 Toronto Centre for Liver Disease, University of Toronto, Toronto, Ontario, Canada.; 7 Department of Medicine, Keck School of Medicine of the University of Southern California, Los Angeles, California, USA.; 8 Department of Medicine, UCSF, San Francisco, California, USA.; 9 University of Pittsburgh Graduate School of Public Health, Pittsburgh, Pennsylvania, USA.; 10 Cleveland Clinic Pediatric Institute, Cleveland, Ohio, USA.; 11 Department of Pediatrics, UCSF, San Francisco, California, USA.; 12 The Hospital for Sick Children and Department of Paediatrics and University of Toronto, Toronto, Canada.; 13 Department of Pediatrics, University of Texas Southwestern Medical Center, Dallas, Texas, USA.; 14 Department of Internal Medicine, Virginia Commonwealth University, Richmond, Virginia, USA.; 15 Department of Medicine, Beth Israel Deaconess Medical Center, Boston, Massachusetts, USA.; 16 Baruch S. Blumberg Institute, Doylestown, Pennsylvania, USA.; 17 Department of Pathology and Laboratory Medicine, University of Pennsylvania Perelman School of Medicine, Philadelphia, Pennsylvania, USA.; 18 Department of Medicine, University of Texas Southwestern Medical Center, Dallas, Texas, USA.; 19 Laboratory of Pathology, National Cancer Institute, Bethesda, Maryland, USA.; 20 Department of Internal Medicine, University of Michigan, Ann Arbor, Michigan, USA.

**Keywords:** Hepatology, Infectious disease, Adaptive immunity, Fibrosis, Innate immunity

## Abstract

Studies of human hepatitis B virus (HBV) immune pathogenesis are hampered by limited access to liver tissues and technologies for detailed analyses. Here, utilizing imaging mass cytometry (IMC) to simultaneously detect 30 immune, viral, and structural markers in liver biopsies from patients with hepatitis B e antigen^+^ (HBeAg^+^) chronic hepatitis B, we provide potentially novel comprehensive visualization, quantitation, and phenotypic characterizations of hepatic adaptive and innate immune subsets that correlated with hepatocellular injury, histological fibrosis, and age. We further show marked correlations between adaptive and innate immune cell frequencies and phenotype, highlighting complex immune interactions within the hepatic microenvironment with relevance to HBV pathogenesis.

## Graphical abstract

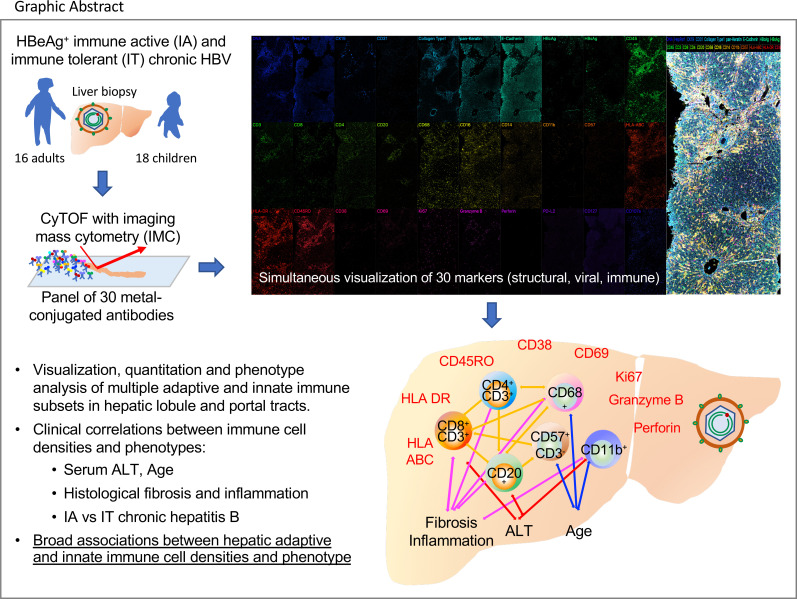

## Introduction

Hepatitis B virus (HBV) is a hepatotropic DNA virus that causes acute and chronic necroinflammatory liver disease (1–3). Globally, more than 250 million people have chronic hepatitis B, with significant morbidity and mortality due to cirrhosis and liver cancer. The natural history of chronic hepatitis B comprises clinical phases defined by dynamic fluctuations over time in serum levels of HBV DNA and alanine aminotransferase (ALT) activity (4) with or without hepatitis B e antigen (HBeAg), a viral marker associated with higher viral titer, greater risk for liver cancer, and immune tolerizing effect (5–9). While a minority of patients present with high titer viremia and normal ALT in an apparent immune-tolerant (IT) phase, others can present in immune-active (IA) phase, with elevated ALT reflecting hepatocellular injury, followed by clinical resolution to an inactive phase or persistent disease with recurrent flares of ALT and viremia, as well as liver disease progression (4–6).

The underlying mechanisms that drive these clinical transitions and hepatocellular injury are not well understood. While HBV is not directly cytopathic, HBV-specific adaptive immune response is critical for virus control, with antiviral CD8^+^ T cells mediating direct cytopathic and noncytopathic virus control as shown in animal models (1–3). In patients with chronic hepatitis B, however, both virus-specific and generalized adaptive immune responses are dampened through multiple regulatory and metabolic pathways (9–14), thereby suggesting alternate modes of liver disease pathogenesis. However, due to safety considerations and low patient acceptance for invasive liver biopsy, as well as limited technologies to comprehensively examine minute liver biopsy tissues ex vivo (15), most studies of human HBV immune pathogenesis have focused on the peripheral blood compartment (9–14). Furthermore, while other approaches using immune cells collected from hepatic perfusates or processed liver tissue can provide key insights, they cannot directly visualize the hepatic parenchyma and identify immune subsets directly infiltrating the hepatic lobule or portal tract (PT) (16–18). Thus, there is a gap in our knowledge of the immune microenvironment in HBV-infected liver and age-dependent differences in HBV immune pathogenesis (19).

Recent emergence of high-dimensional tissue imaging approaches enables visualization of a complex tissue microenvironment with unprecedented level of details (20, 21). In particular, in imaging mass cytometry (IMC), formalin-fixed paraffin-embedded (FFPE) tissues are simultaneously stained with a panel of 30–40 antibodies tagged with rare earth metal isotopes and successively ablated by high-resolution laser for cytometry TOC (CyTOF) analysis of mass signal per μm^2^ of ablated tissue, with downstream image reconstruction based on mass signal and spatial information. To date, the use of IMC has yielded insights to human endocrine pancreas in type 1 diabetes, cancers, and fetal immune development (22–28); however, to our knowledge, no studies of HBV-infected liver or viral markers have been performed, to date.

In this study, we took advantage of IMC technology and available FFPE liver tissues from a well-characterized cohort of adults and children with HBeAg^+^ IT or IA chronic hepatitis B (5, 29), to better define their intrahepatic immune microenvironment and immune interactions relative to clinical parameters. Our panel of 30 antibodies enabled simultaneous detection of CD4^+^ and CD8^+^ T cells, B cells, hepatic monocyte/macrophage populations, and NK cells, as well as HBV antigens, hepatocytes, and other hepatic portal and lobular structures. This highly multiplexed visualization identified greater adaptive and innate immune frequency and activation in patients with IA compared with IT chronic hepatitis B, with significant clinical correlations. We further demonstrate remarkable associations between hepatic adaptive and innate immune frequencies and phenotypes, with a high density of multiple immune subsets in the portal triad of HBV-infected individuals.

## Results

### Study population with HBeAg^+^ IA and IT chronic hepatitis B.

We included 34 HBeAg^+^ subjects with IA (28 total; 14 children, 14 adults) or IT (6 total; 4 children, 2 adults) chronic hepatitis B (Supplemental Table 1), with available FFPE liver biopsy slides from the National Institutes of Diabetes and Digestive and Kidney Diseases–sponsored (NIDDK-sponsored) Hepatitis B Research Network (HBRN). For comparisons, 10 noninfected controls (NC) undergoing hepatic resection for metastatic liver masses were included, as described in Methods. HBV-infected subjects were mostly of Asian ancestry, with an age range of 2–51 years. Consistent with clinical chronic hepatitis B phenotype definitions (9, 29), ALT values were higher in IA compared with IT subjects, with a wide range (11–539 U/L). Three subjects were in ALT flare, with ALT levels above 10 times the upper limit of normal (ULN) at 446, 371, and 539 U/L, whereas 1 subject (IA-A1) with low ALT (21 U/L) had an ALT flare 8 months before the liver biopsy. There were no significant differences between IT and IA subjects in age, sex, race, or HBV genotype. Consistent with HBeAg^+^ status, both IA and IT subjects showed high serum HBV DNA levels (median 8.2 log IU/mL). These 34 HBeAg^+^ chronically HBV-infected adults and children with high-level viremia and varied serum ALT levels provided the basis for our analyses.

### Structural, viral, and immune components are readily visualized in liver tissues by IMC.

**Figure 1:**
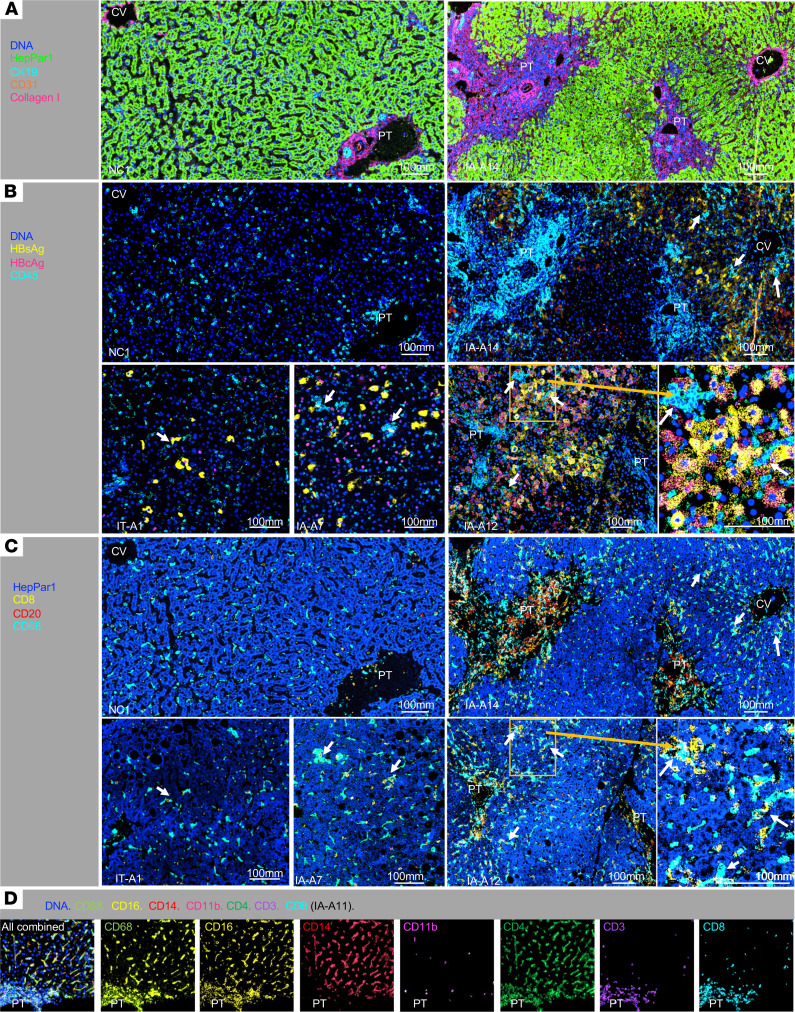
Visualizing multiple structural, immune, and viral markers in HBV-infected livers by image mass cytometry. (**A**) Detection of hepatic structures with nuclear DNA (blue), HepPar1**^+^** hepatocytes (green), CK19**^+^** bile ducts (cyan), CD31**^+^** endothelial cells (orange), and collagen I (pink) in a representative noninfected control (NC1) and chronic hepatitis B (CHB) IA (IA-A6) subject. Portal tract (PT) was defined by the presence of portal triad including CK19**^+^** bile ducts, CD31**^+^** hepatic artery, and portal vein with surrounding collagen I. Central vein (CV) was defined as a vascular structure with surrounding collagen without bile duct or hepatic artery. (**B**) Detection of viral and immune markers including HBsAg (yellow), HBcAg (pink), and CD45**^+^** immune cells (cyan) with the same regions as **A** for NC1 and IA-A14, and additional CHB subjects (IT-A1, IA-A8, and IA-A12) with various patterns of HBsAg and HBcAg expression. White arrows indicate clusters of CD45^+^ immune cells close to hepatocytes expressing HBsAg and/or HBcAg. Orange inlay for IA-A12 provides a higher power view. (**C**) Detection of CD8^+^ T cells, CD20^+^ B cells, and CD68^+^ Kupffer cells. Regions in **B** are shown here with CD8 (yellow), CD20 (red), CD68 (cyan), and HepPar1 (blue), with increased CD20^+^ B cells, yellow CD8^+^ T cells, and cyan CD68^+^ cells in portal tracts (especially in IA-A14), and more diffuse lobular detection of cyan CD68^+^ cells. White arrows highlight CD45^+^ immune clusters near hepatocytes expressing HBsAg and/or HBcAg in **B**, with both yellow CD8^+^ T cells and cyan CD68^+^ Kupffer cells located in close contact. (**D**) Representative distribution for innate and adaptive immune markers in the liver. Colocalization of CD68, CD16, and CD14 in hepatic lobular region, with a similar pattern for CD4 expression (without associated CD3 expression). Relative paucity of CD14 expression compared with other markers (e.g., CD3, CD8, CD4, CD68, CD16) is noted in portal tract (PT).

We developed an antibody panel for 30 markers (Supplemental Table 2 and Supplemental Figure 1) for IMC detection of hepatic structural, viral, and immune markers, as described in Methods. We first wanted to determine if we can identify various structural, viral, and immune markers in liver tissues by IMC. As described in Methods, regions of interest (ROIs) in each slide were randomly selected based on bright-field images and acquired by IMC, followed by image analysis and cell segmentation (Supplemental Figures 1 and 2). As shown in Figure 1, overlay of select markers enabled visualization of hepatic structures, including hepatocytes staining for antihepatocyte-specific antigen HepPar1, PT, with type I collagen, portal triad (CK19^+^ bile duct, CD31^+^ hepatic artery, portal vein), and central veins (CV) (Figure 1A), as well as HBV antigens and immune cells (Figure 1, B–D, and Supplemental Figure 3).

As shown in Figure 1B and Supplemental Figure 3B, varying degrees of cytoplasmic hepatitis B surface antigen (HBsAg) and nuclear and/or cytoplasmic hepatitis B core antigen (HBcAg) expression were detected in isolated or multiple contiguous hepatocytes in liver tissues from HBV-infected IA/IT but not NC subjects. Liver tissues from IT subjects did not show higher HBsAg and/or HBcAg expression compared with IA subjects, whereas intense cytoplasmic HBsAg expression was detected in several IA subjects (e.g., IA-P6, IA-A8, IA-A11, IA-A12). HBcAg expression was largely nuclear in IT subjects, whereas cytoplasmic (as well as nuclear) HBcAg expression was detected in IA subjects, as previously reported (30).

Portal enrichment for immune cells was noted in IA subjects, based on cells expressing the leukocyte common antigen CD45 (Figure 1B and Supplemental Figure 3). As shown for IA-A14 and IA-A12 (Figure 1, B and C), portal CD45^+^ immune infiltrates included CD8^+^ T cells, CD20^+^ B cells, and CD68^+^ cells (Figure 1, B and C). In the hepatic lobules, there were also CD45^+^ immune clusters consisting of both CD8^+^ and CD68^+^ cells, some in close proximity to hepatocytes expressing HBsAg and/or HBcAg (highlighted by white arrows in Figure 1, B and C).

Figure 1D further shows colocalization of CD68, CD16 (Fcγ receptor III), and CD14 (LPS receptor) expression in lobular regions, consistent with their coexpression reported in hepatic Kupffer cells and monocytes (31–33). To simplify, we refer to CD45^+^ cells expressing CD68, CD16, and/or CD14 collectively as Kupffer cells in this study. Notably, while CD4 is generally a marker of CD4^+^CD3^+^ T cells, most lobular CD4 expression was colocalized with CD68, CD16, and/or CD14 but not CD3, indicating that these are Kupffer cells with CD4 coexpression (34). We further noted cells expressing CD11b — a β-2 integrin expressed in myeloid cells including macrophages, DCs, and neutrophils (35, 36) — with CD68 and CD16 coexpression, and we refer to them as a distinct CD11b^+^ hepatic innate immune subset. Expression of maturation marker CD57 in CD45^+^CD3^–^ cells was used to define a mature subset of NK cells. These findings show that IMC can simultaneously visualize structural, viral, and immune markers in the liver with unprecedented levels of detail and complexity.

### Hepatic immune cells can be segregated from non–immune cells and quantified for clinical correlations.

**Figure 2:**
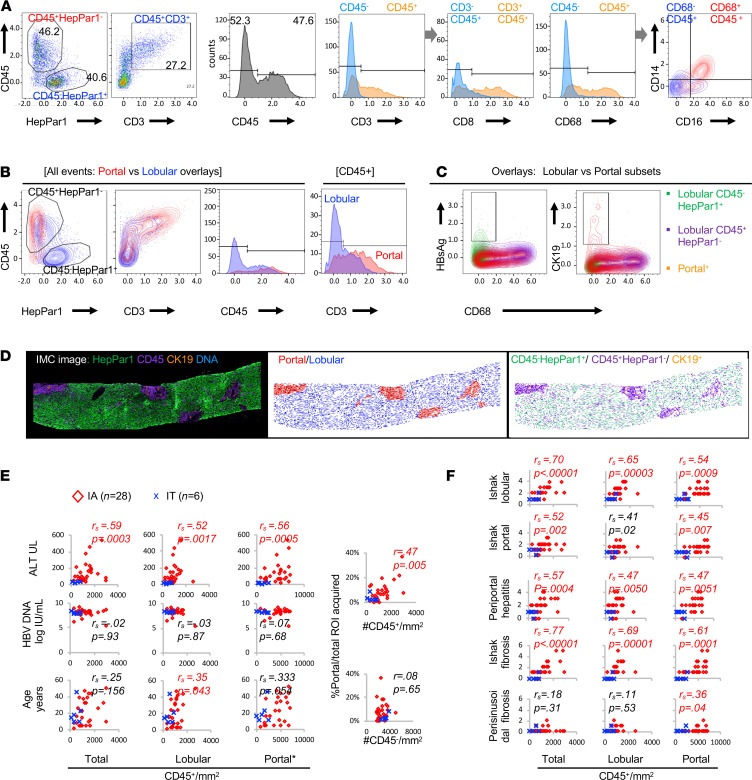
Portal and lobular distribution of CD45^+^ immune cells quantified in chronic hepatitis B (CHB) and control liver tissues by IMC. (**A**) Acquired IMC images are visualized in representative pseudocolor plots and histogram with overlays to show that HepPar1^+^ events can be gated separately from CD45^+^ events, and that CD45^+^ events are enriched for immune markers (e.g., CD3, CD8, CD68). Far right contour plot overlay shows that CD68^+^CD45^+^ gate (red) is enriched for concurrent CD16/CD14 expression, compared with CD68^–^CD45^+^ gate (blue). (**B**) Representative contour plot and histogram overlays provide comparisons for portal (red) versus lobular (blue) detection of HepPar1, CD45, and/or CD3 expression, with expected lobular but not portal detection for HepPar1^+^ hepatocytes and both lobular and portal detection for CD45^+^ or CD3^+^ immune cells. (**C**) Overlay of lobular CD45^–^HepPar1^+^ (green), lobular CD45^+^HepPar1^–^ (purple), and all portal (orange) events show HBsAg expression limited to CD45^–^HepPar1^+^ cells, CK19 expression limited to portal cells, and CD68 expression in both lobular CD45^+^HepPar1^–^ immune cells and portal cells. (**D**) Tissue image reproduced with portal and/or lobular location of bile ducts, hepatocytes, and immune cells by applying *x*/*y* spatial coordinates to analyzed IMC data. (**E**) Scatter plots comparing %portal/total area, ALT, HBV DNA, and age relative to total, lobular, and portal CD45^+^ cell density per mm^2^ for 28 IA (red diamond) and 6 IT (blue X) subjects. A single outlier with portal CD45^+^ immune cell density at 43,365 was not shown graphically, although it was included in calculating the Spearman’s correlation and *P* values (shown in red fonts for *P* < 0.05). (**F**) Scatter plots comparing histological scores (Ishak lobular inflammation, Ishak portal inflammation, Ishak periportal hepatitis [piecemeal necrosis], Ishak fibrosis, and perisinusoidal fibrosis scores) with total, lobular, and portal CD45^+^ cell density per mm^2^ for 28 IA (red diamond) and 6 IT (blue X) subjects, with Spearman’s correlations and *P* values.

Using the imaging and cell segmentation strategy as described in Methods and Supplemental Figures 1 and 2, we next examined if hepatic immune and non–immune cells can be graphically visualized for quantitative analyses. As shown in Figure 2A, distinct CD45^+^HepPar1^–^ “immune” and CD45^–^HepPar1^+^ “hepatocyte” populations could be separated in scatterplots, histogram overlays, and 2-dimensional images, showing greater enrichment for immune markers (e.g., CD3 and CD68) in CD45^+^ immune compared with CD45^–^ nonimmune compartment, as well as enrichment for CD8 expression in CD3^+^CD45^+^ T cell compared with the non–T cell compartment. Coexpression of CD16 and CD14 was noted in CD68^+^CD45^+^ cells (Figure 2A, far right), thereby recapitulating CD68, CD16, and CD14 coexpression in Kupffer cells in IMC images in Figure 1D.

As for their portal and lobular location, CD45^+^HepPar1^–^ or CD45^+^CD3^+^ immune subsets were detected in both lobular (shown in blue) and portal (shown in red) locations, as expected (Figure 2B). A distinct CD45^–^HepPar1^+^ population was detected exclusively in lobular but not portal locations, consistent with the lobular location of hepatocytes. As expected, HBsAg expression was limited to lobular CD45^–^HepPar1^+^ hepatocytes, whereas expression of CK19 (a marker for biliary epithelial cells) was limited to cells from the PT (Figure 2C). As shown in Figure 2D, our portal and lobular masking strategy reproduced the IMC image with lobular CD45^–^HepPar1^+^ hepatocytes and portal CK19^+^ bile duct epithelial cells, with both portal and lobular distribution of CD45^+^HepPar1^–^ immune cells. Collectively, these results support our IMC analytic pipeline in distinguishing hepatic portal and lobular immune subsets for further analyses.

Based on our analytic approach, a median of 1990 CD45^+^ immune cells were counted from each acquired ROI per subject (median ROI, 1.9 mm^2^) (Supplemental Table 3, A and B). Since acquired ROI and relative percentage of portal region varied between groups, hepatic CD45^+^ immune cells were examined as a concentration or density per total, lobular, or portal areas in mm^2^ of acquired ROI. As shown in Supplemental Table 3C, this resulted in median hepatic CD45^+^ cell density of 964/mm^2^ ROI overall, with significant 2- to 3-fold differences between IA and IT subjects in total, lobular, and portal regions. Notably, total and portal CD45^+^ cell densities were significantly greater in NC compared with IT subjects. The differences between NC and IT subjects likely reflect underlying metastatic cancer in our noninfected NC subjects (37), although we only examined non–tumor tissues from NC subjects. Based on these findings, further comparisons between groups were focused on IA and IT — but not NC subjects thereafter.

As for their clinical correlations, IMC-derived total, lobular, and portal CD45^+^ cell densities in HBV-infected subjects correlated significantly with serum levels of ALT but not HBV DNA, as shown in Figure 2E. Positive correlation was also noted between age and lobular CD45^+^ cell density (*r*_s_ = 0.35, *P* = 0.043). Furthermore, area within acquired ROI as a measure of portal expansion correlated positively with total CD45^+^ immune but not CD45^–^ non–immune cell density. Finally, IMC-derived hepatic CD45^+^ cell density showed highly significant positive correlations with histological inflammation and fibrosis scores (38, 39) derived from entire liver biopsies by clinical pathologists (Figure 2F). Thus, our IMC analytic strategy enabled quantitation of hepatic immune cells with significant clinical correlations.

### Simultaneous IMC analysis defines a quantitative hierarchy and associations among multiple hepatic adaptive and innate immune subsets.

**Figure 3:**
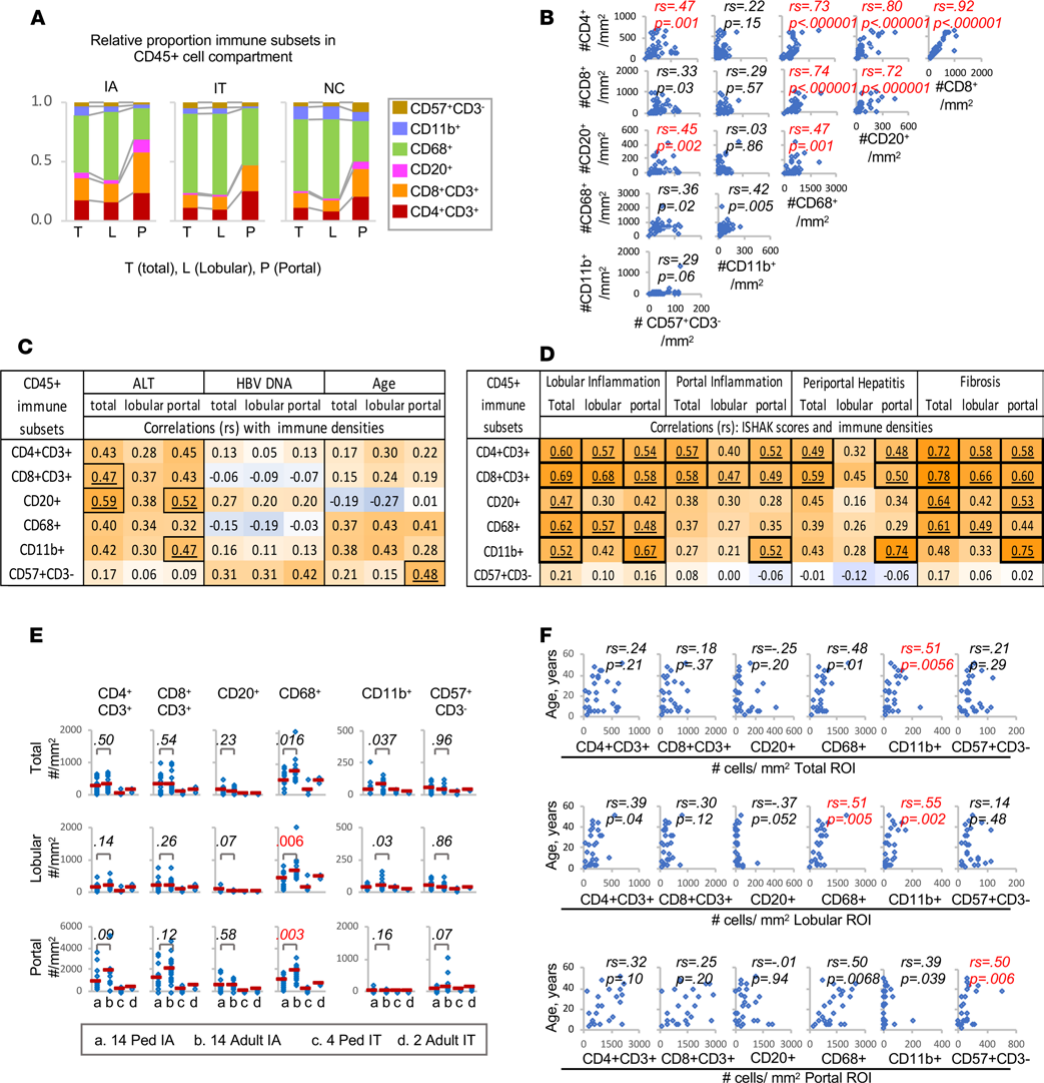
CD45^+^ adaptive and innate immune cells in the liver in HBV-infected and uninfected subjects. (**A**) Stacked bar graphs show relative proportions of adaptive and innate immune subsets within total, lobular, and portal regions. (**B**) Hepatic concentrations of adaptive and innate immune subset per mm^2^ ROI are compared with each other, with Spearman’s correlation coefficients and *P* values shown in right upper part of each scatterplots. *P* < 0.0033 were considered significant and highlighted in red font. (**C** and **D**) Heatmaps showing Spearman’s correlation coefficients (*r*_s_) comparing total, lobular, and portal hepatic immune cell concentrations (in number of cells/mm^2^) to serum ALT (U/L), HBV DNA (log HBV DNA IU/mL), and histological Ishak scores among 28 IA and 6 IT subjects, and with %portal/total ROI as a measure of portal expansion. Correlations associated with *P* < 0.0083 are highlighted by bold font and black border. (**E**) Dot plots comparing 14 pediatric IA, 14 adult IA, 4 pediatric IT, and 2 adult IT subjects with chronic hepatitis B for median hepatic CD45^+^ immune cell density/mm^2^ in total, lobular, and portal region, with error bars indicating 25% and 75% IQRs. *P* values were calculated by Mann-Whitney *U* test. *P* < 0.00625 were considered significant and highlighted in red font. (**F**) Comparisons between age and total, lobular, and portal hepatic immune densities in 28 IA subjects, with Spearman’s correlation (*r*_s_) and *P* values shown in red font for *P* < 0.00625.

We next examined the distribution of various adaptive and innate immune subsets within the CD45^+^ immune compartment. As shown in Figure 3A and Table 1, Kupffer cells showed total, lobular, and portal predominance in all groups, followed by CD8^+^ and CD4^+^ T cells, with the exception for IA subjects in whom CD8^+^ T cells were the most predominant among portal immune subsets. Overall, adaptive immune cells showed marked enrichment in portal compared with lobular region (e.g., up to 19- to 20-fold for CD20^+^ B cells). Portal enrichment was also noted for CD68^+^ Kupffer cells and CD57^+^CD3^–^ NK cells, although to a lesser degree compared with adaptive immune cells. By contrast, CD11b^+^ cells showed greater lobular enrichment. Notably, hepatic densities of all 3 adaptive immune subsets showed significant correlations with each other — and with hepatic densities of CD68^+^ Kupffer cells and/or CD57^+^CD3^–^ NK cells (Figure 3B). Collectively, these findings highlight immune hierarchy, as well as remarkably close interplays among adaptive and innate immune subsets within the hepatic microenvironment.

### Hepatic lobular and portal densities of adaptive and innate immune subsets correlate with clinical parameters.

We then directly compared the hepatic immune densities between IA and IT subjects. Not surprisingly, the total, lobular, and portal hepatic densities for most immune subsets examined were greater in IA compared with IT subjects, with statistical significance reached for total, lobular, and portal CD20^+^ B cells, as well as portal CD4^+^ T cells, CD8^+^ T cells, and CD68^+^ Kupffer cells (Table 1, far right). Furthermore, as shown in Figure 3C, most adaptive and innate immune cell densities correlated positively with serum ALT, reaching statistical significance for total and/or portal CD8^+^ T cells, CD20^+^ B cells, and CD11b^+^ cells. Significant positive correlation was also noted between age and portal CD57^+^CD3^–^ NK cell density, perhaps reflecting an age-dependent increase reported in mature CD57^+^ NK cells (40) but not between HBV DNA levels or any of the hepatic immune densities. Hepatic densities of most immune subsets showed significant positive correlations with histological scores of hepatic inflammation and/or fibrosis, consistent with broad immune participation in HBV-associated liver inflammation and fibrogenesis (Figure 3D). Thus, hepatic immune cell densities were broadly increased in IA compared with IT subjects, in direct correlations with hepatocellular injury, inflammation, and fibrosis.

### Age is positively correlated with hepatic innate immune cell densities in HBV-infected patients.

Inclusion of HBV-infected children and adults provided an opportunity to examine the potential effect of age on hepatic immune infiltrates, as previously suggested (41). As shown in Figure 3E, hepatic CD68^+^ Kupffer cell densities were significantly lower in children compared with adults with IA chronic hepatitis B, without significant differences reached for other immune subsets. A similar pattern was noted for IT subjects, although sample sizes were too small for statistical comparison. Among IA subjects, age showed significant positive correlations with innate immune cell densities (Figure 3F).

### Hepatic immune subsets display distinct activation, memory, and effector phenotypes with greater activation but not effector phenotypes in IA compared with IT subjects.

**Figure 4:**
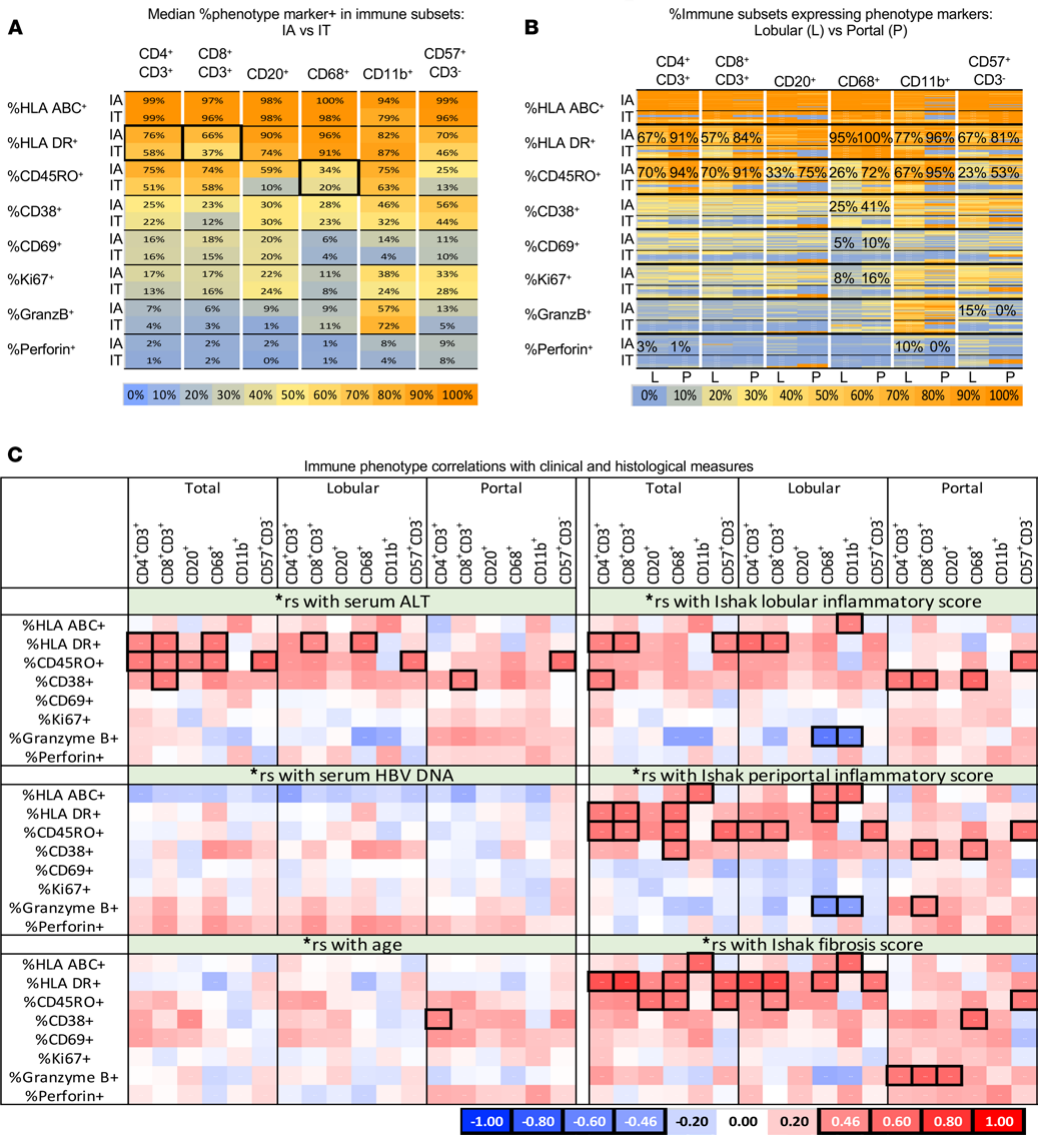
Phenotype of CD45^+^ immune subsets in the liver. (**A**) Greater HLA DR and/or CD45RO expression by hepatic immune subsets from IA compared with IT subjects. Median percentages of cells expressing various phenotype markers within each immune subset are shown as a heatmap from IA (*n* = 28) and IT (*n* = 6) groups. Significant percentage differences between IA and IT subjects (with *P* values < 0.00625 by Mann-Whitney *U* test) are highlighted in bold font with thick borders. (**B**) Portal enrichment for immune subsets with activation phenotype. Heatmap represents percentages of lobular (L) and portal (P) immune subsets that express various phenotype markers from individual IA and IT subjects, with median percentages indicated for significant phenotype differences between lobular and portal immune subsets (*P* < 0.00625 by nonparametric signed-rank test). (**C**) Immune phenotype correlations with clinical and histological measures. Heatmaps show Spearman’s correlation coefficients (*r_s_) comparing serum ALT, HBV DNA, and age with percentages of cells expressing various markers in 6 CD45^+^ immune subsets. Correlations associated with significant *P* values below 0.0625 are indicated with thick/black borders. Positive and negative values of correlation coefficient corresponding to the red to blue colors are indicated for reference, with cutoff *r*_s_ values (± 0.46) shown. *P* values associated with the correlation coefficients are provided in Supplemental Figure 4.

We then examined if hepatic immune activation, memory, and/or effector phenotypes correlate with clinical or virological status in HBV-infected patients using the following markers: HLA class I (HLA ABC), HLA class II (HLA DR), memory (CD45RO), activation (HLA DR, CD38 and CD69), proliferation (Ki67), and cytolytic effector molecules (granzyme B, perforin). As shown in Figure 4A, hepatic immune subsets were most enriched for HLA ABC and HLA DR expression, followed by CD45RO and CD38, with less expression of CD69, Ki67, granzyme B, and perforin. CD11b^+^ cells also showed prominent granzyme B expression, as reported in Kupffer cells in patients with chronic hepatitis (42).

As shown in Figure 4B and Supplemental Figure 4B, portal immune subsets tended to be more activated than their lobular counterparts in IA subjects, reaching statistically significant differences for HLA DR and CD45RO expression for most immune subsets, as well as CD38, CD69, and Ki67 expression in CD68^+^ Kupffer cells. By contrast, granzyme B and perforin showed significantly greater lobular than portal enrichment in CD4^+^ T cells, CD11b^+^ cells, and CD57^+^CD3^–^ cells.

Comparisons between IA and IT subjects showed greater immune activation in IA compared with IT subjects based on HLA DR expression in CD4^+^ (76% versus 58%, *P* = 0.004) and CD8^+^ T cells (66% versus 37%, *P* = 0.002), as well as CD45RO expression in CD68^+^ Kupffer cells (34% versus 20%, *P* = 0.006) (Figure 4A). Differential CD38 expression between IA and IT subjects was also noted for portal CD4^+^ T cells (28% versus 10%, *P* = 0.003) and CD68^+^ Kupffer cells (41% versus 11%, *P* = 0.005). Immune subsets from IA and IT subjects did not differ significantly in their granzyme B or perforin expression. Thus, hepatic immune subsets from IA subjects showed a greater activation phenotype than those from IT subjects, without differential expression of cytolytic effector molecules.

### Hepatic immune subsets in patients with chronic hepatitis B display distinct phenotypes that correlate with clinical, demographic, and histological parameters.

The percentages of cells expressing various phenotype markers within each immune subset were correlated with clinical and histological parameters, with correlation coefficients displayed as a heatmap in Figure 4C and select scatterplots shown in Supplemental Figure 4C. As shown, ALT showed significant positive associations with activation and/or memory phenotype (e.g., HLA DR, CD45RO, CD38) in most immune subsets. As for HBV DNA titers, correlations were largely negative for HLA ABC expression but were positive for granzyme B and perforin expression, although statistical significance was not reached for any subsets. Age tended to correlate positively with CD45RO, CD38, and CD69 expression in adaptive immune subsets, with statistical significance reached for CD38 expression in portal CD4^+^ T cells.

Figure 4C shows that histological inflammation and fibrosis scores correlate positively with activation and memory phenotype of most hepatic immune subsets. By contrast, histological inflammation and fibrosis scores correlated negatively with granzyme B expression in most lobular immune subsets (especially CD68^+^ and CD11b^+^ cells) and positively with granzyme B expression in most portal immune subsets (especially T and B cells). Furthermore, comparisons of activation and effector phenotype between the adaptive and innate immune subsets showed broad correlations among various adaptive and innate immune subsets (Supplemental Figure 4D). These findings highlight distinct phenotypes of hepatic immune subsets and their interactions in chronic hepatitis B with clinical correlations.

### Cluster analysis of IMC data identifies immune subsets that correspond to various adaptive and innate immune subsets, with clinical correlations.

**Figure 5:**
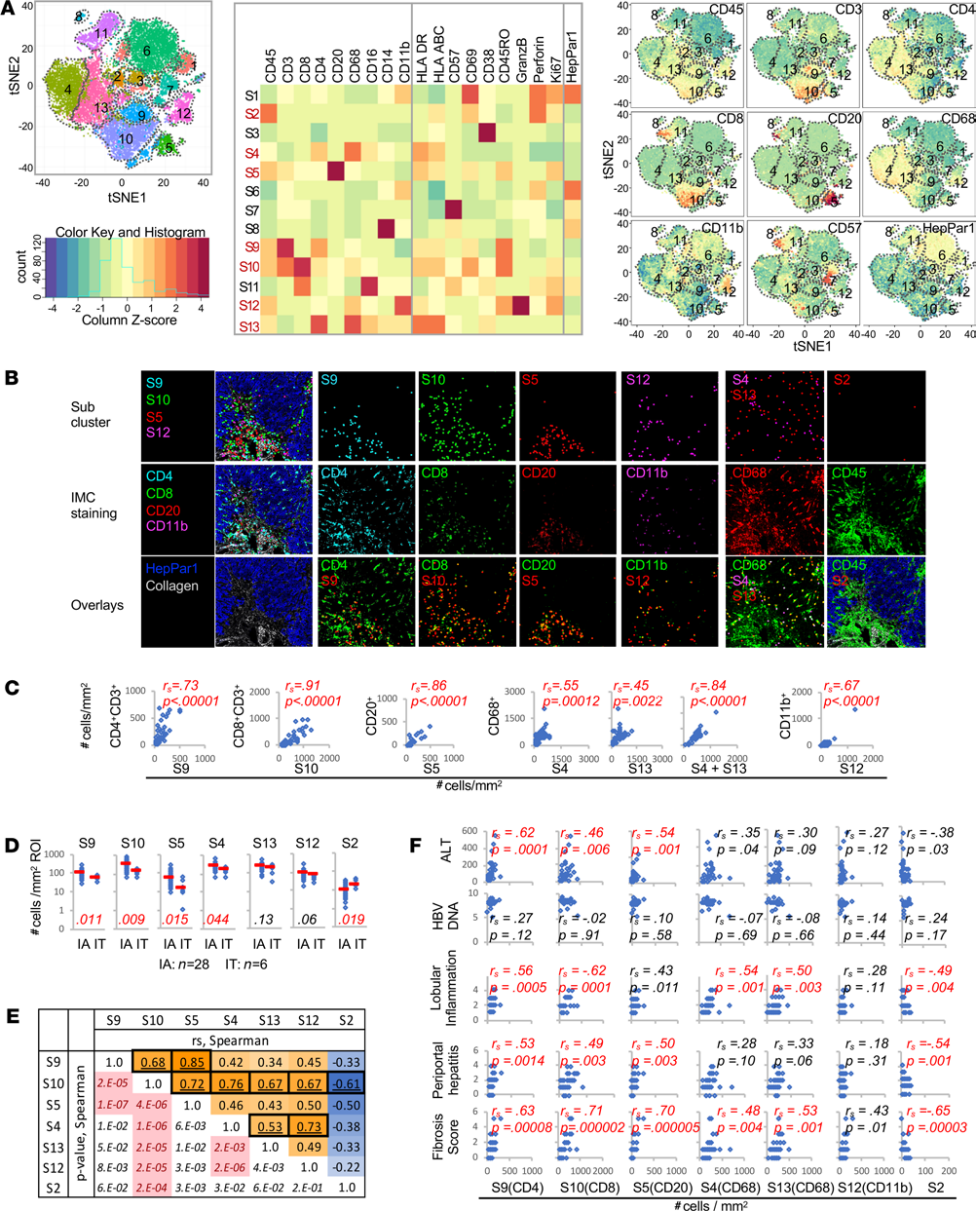
PhenoGraph analysis identifies distinct immune subclusters that correlate with serum ALT. (**A**) PhenoGraph analysis of IMC data shown as: (left) a tSNE plot with 13 subclusters (S1–S13); (middle) a heatmap displaying expression of cellular markers; (right) 9 representative tSNE plots showing various marker expression. (**B**) Representative IMC images with PhenoGraph subcluster events shown as distinct dots (top); select antibody staining patterns in IMC images (middle); overlays of subcluster dots in red onto individual markers in green, with yellow color representing colocalization (bottom). Three images on the far left and bottom far right show background of HepPar1 in blue and collagen I in light gray to indicate hepatic architecture. (**C**) Scatter plots comparing cellular densities (cells/mm^2^ ROI) for immune subclusters on the *x* axis and manually gated CD45^+^ immune subsets on the *y* axis for 28 IA, 6 IT, and 10 NC subjects. Spearman’s correlation coefficients (*r*_s_) and *P* values are shown, with red font for *P* < 0.05. (**D**) Dot plots comparing hepatic densities of CD45-enriched subclusters between 28 IA and 6 IT subjects. Median values shown as red horizontal bars. *P* values by Mann-Whitney *U* tests with values < 0.00625 highlighted in red font. (**E**) Upper right half of the table shows Spearman’s correlation coefficients between immune subcluster densities as a heatmap (positive correlations in orange and negative correlations in blue) with corresponding *P* values in left bottom half, with significant *P* values highlighted in pink with red font (*P* < 0.00238 considered significant). (**F**) Scatter plots comparing subcluster densities (cells/mm^2^ ROI) on the *x* axis and serum ALT levels, HBV DNA, and Ishak histological scores on the *y* axis. Spearman’s correlation coefficients (*r*_s_) and *P* values are shown. Correlations with *P* < 0.0071 were considered significant and highlighted in red.

We next applied the clustering algorithm PhenoGraph to IMC data as described in Methods, in order to identify immune subsets in a less biased manner. Among 13 subclusters with distinct spatial separation and phenotype characteristics (Figure 5A, left panel), 7 “immune” subclusters were enriched for CD45 expression and additional immune markers, but not HepPar1 (Figure 5A, middle and right panels). These included: 3 “adaptive” subclusters: S9 (enriched for CD3 and CD4), S10 (enriched for CD3 and CD8), and S5 (enriched for CD20); 3 “innate” subclusters: S4/S13 (both enriched for CD68, CD16, CD4) and S12 (enriched for CD11b, CD68 and granzyme B); and an S2 subcluster enriched for perforin and CD69 but without other distinguishing markers. None of the CD45^+^ immune subclusters were enriched for CD57^+^CD3^–^ NK cells. As shown in Figure 5B and Supplemental Figure 5C, events defined by the subclusters also colocalized with manually gated immune subsets as follows: S9 with CD4; S10 with CD8; S5 with CD20; S12 with CD11b; S4/S13 with CD68; and S2 with CD45. Accordingly, there were significant positive correlations between hepatic densities for the immune subclusters and corresponding subsets identified by manual gating (Figure 5C), except for the S2 subcluster, which correlated inversely with hepatic densities of most immune subsets examined (Supplemental Figure 5D).

As shown in Figure 5D, CD8 subcluster (S10) and Kupffer cell subclusters (S4 and S13) showed the highest hepatic densities, followed by S9, S12, and S5 subclusters enriched for CD4^+^ T cells, CD11b^+^ cells, and CD20^+^ B cells, respectively. As expected, hepatic densities of most immune subclusters were greater in IA compared with IT subjects. Furthermore, hepatic densities of most immune subclusters showed significantly positive correlations with each other, with the exception of S2 with negative correlations (Figure 5E). Finally, as shown in Figure 5F, hepatic densities for all 3 adaptive immune subclusters (S9, S10, and S5) correlated positively with serum ALT, hepatic inflammation, and hepatic fibrosis scores — but not HBV DNA levels or age (data not shown). Significant positive correlations with hepatic inflammation and fibrosis were also noted for innate subclusters S4 and S13, with negative correlations for S2. Collectively, results of these computational analyses further support our findings correlating hepatic adaptive and innate immune subsets in HBV pathogenesis.

## Discussion

Liver disease in HBV infection is immune mediated. While HBV-specific CD8^+^ T cells are key effectors that mediate both hepatocellular injury and virus control (1–3), a paradigm has been emerging in the past decade, positing that multiple immune subsets participate in HBV-associated liver disease pathogenesis (9–14). There is also some controversy in distinguishing between antiviral immunity and inflammation relevant for clinical phases of chronic hepatitis B (7, 19). In this study, we applied a highly multiplexed IMC approach to provide potentially novel comprehensive visualization of HBV-infected liver tissues with simultaneous quantitative and phenotypic analyses of multiple immune subsets that correlated with clinical parameters.

A key finding in our study is that hepatic densities of most adaptive and innate immune subsets showed remarkably close correlations with each other (e.g., between CD4^+^ and CD8^+^ T cells, T cells and B cells, T cells and Kupffer cells, B cells and Kupffer cells), with further correlations in their activation and/or effector phenotypes. Furthermore, hepatic densities of most adaptive and innate immune subsets correlated significantly with serum ALT, a clinical measure of hepatocellular injury, and with histological fibrosis, as well as inflammation. These findings suggest close interplays among hepatic immune subsets that are collectively induced and contribute to liver disease pathogenesis.

Our findings also highlighted distinct immune differences between patients with clinically defined IA and IT chronic hepatitis B. First, hepatic immune densities were significantly greater in IA compared with IT subjects — for T cells and B cells, as well as for Kupffer cells. Second, hepatic T cells and Kupffer cells showed a greater activation phenotype in IA compared with IT subjects, but without differential expression of effector molecules granzyme B or perforin. Third, hepatic densities, as well as HLA DR, CD45RO, and/or CD38 expression by most immune subsets, showed significant positive correlations with serum ALT, as well as histological inflammation and fibrosis. These findings support the use of ALT elevation as a marker for “immune activation” in chronic hepatitis B. At the same time, “immune activation” in this context represented generalized inflammation involving multiple immune subsets (both adaptive and innate), rather than activation of antiviral effector T cells, which we did not examine in this study but are known to be functionally exhausted in chronic hepatitis B.

Serum ALT level showed significant positive correlation with HLA DR and CD45RO, but not granzyme B expression, by Kupffer cells. On the other hand, granzyme B expression by lobular Kupffer cells and CD11b^+^ cells correlated negatively with histological inflammation, whereas granzyme B expression in portal (but not lobular) T and B cells correlated positively with hepatic fibrosis and/or periportal inflammation. These findings raise the possibility for broad immune participation in hepatocellular injury, including lobular Kupffer cells and CD11b^+^ cells, which may be activated with degranulation of cytolytic effector molecules.

Serum HBV DNA levels did not correlate with hepatic densities or phenotype of most immune cells, including CD8^+^ T cells. This may be due to uniformly high levels of HBV viremia in our HBeAg^+^ patients without sufficient dynamic range in viral titers. Alternatively, this lack of correlation between HBV DNA levels and hepatic immune parameters may represent the ineffective and exhausted antiviral immunity in the setting of generalized inflammation in chronically HBV-infected liver (43, 44).

Clinical phases of chronic hepatitis B are well known to change over time (45, 46). As both children and adults with HBeAg^+^ IA and IT chronic hepatitis B were included in our study, we had an opportunity to examine if age may play a role in hepatic immune composition and phenotype in patients with HBeAg^+^ chronic hepatitis B. In fact, older age was associated with increased hepatic accumulation of Kupffer cells and CD11b^+^ cells, as well as with adaptive immune activation. Thus, age-associated changes in intrahepatic immune subsets might contribute to clinical evolution in chronic hepatitis B.

There are several limitations in our study. First, acquired ROI in our study was small (median 1.9 mm^2^) due to lengthy acquisition time required in IMC. However, our analytic approach yielded results that correlated significantly with clinically relevant parameters (e.g., serum ALT), as well as histological scores derived from the entire biopsy. Second, our cell segmentation strategy focused on small immune cells, rather than large non–immune cells (e.g., hepatocytes), since they require a different cell segmentation strategy. Although beyond the scope of current analysis, further evaluation of non–immune cells and their interplay with immune subsets is warranted in the future — for example, by localizing immune cells (including HBV-specific adaptive immune cells) within the hepatic parenchyma relative to HBV-expressing hepatocytes and their viral antigen expression. Third, regarding our study subjects, we focused on patients with HBeAg^+^ IA and IT chronic hepatitis B, and our HBV NC subjects did not truly represent healthy controls. Nevertheless, our analysis within HBeAg^+^ subjects showed significant immune correlations with serum ALT, age, and histological measures.

Collectively, to our knowledge, these findings provide the first IMC visualization and quantitative analysis of the complex immune landscape in the liver of patients with HBeAg^+^ chronic hepatitis B, showing significant immune correlations with hepatocellular injury, inflammation, and fibrosis in addition to broad immune interplays between the adaptive and innate immune cells. Our study also provides a proof of principle and the foundation to apply highly multiplexed IMC as a tool for more detailed and virus-specific analyses to study HBV, as well as other viral immune pathogenesis in the liver.

## Methods

### Patient liver tissues.

FFPE slides of liver biopsy samples from 34 HBeAg^+^ chronically HBV-infected participants in the NIDDK-supported HBRN were obtained from the NIDDK Central Repository (Frederick, Maryland, USA). These samples were obtained between 2011 and 2016 from 28 IA (14 pediatric, 14 adults) and 6 IT (4 pediatric, 3 adults) HBRN participants with chronic hepatitis B, with associated clinical, virological, and histological parameters as shown in Supplemental Table 1. Liver biopsy slides from HBRN participants were also independently reviewed and graded by HBRN pathologists according to a well-defined scoring system (38, 39). Deidentified liver tissues were obtained from Tumor Tissue and Biospecimen Bank on-site at the University of Pennsylvania from 10 NC who were undergoing hepatic resection of metastatic liver masses and no known history of HBV infection but without otherwise complete clinical or demographic information. Of note, NC tissue slides were selected from uninvolved non–tumor tissue regions.

### Antibodies.

Supplemental Table 2 shows the panel for 30 markers, including 17 metal-conjugated antibodies plus Iridium 191/193 that were purchased directly from Fluidigm; with additional 12 antibodies from Santa Cruz Biotechnology Inc., BioLegend, LSBio, Abcam, and R&D Systems that were metal conjugated in house using Maxpar X8 Antibody Labeling Kits (Fluidigm) according to the manufacturer’s instructions. Antibody specificities in IMC were validated as previously described (22), selecting antibodies that showed specificities in immunofluorescence, flow cytometry, and/or mass cytometry and testing them in various titrations to stain FFPE liver (and other control tissues) in IMC. For HBsAg and HBcAg staining, FFPE liver tissues from known HBV-infected and uninfected subjects were stained with various antibody titrations, in addition to HBV Core Antigen Positive Control Slide from MilliporeSigma (catalog 216S). Collectively, the antibodies defined (a) hepatic structural markers such as hepatocytes (HepPar1^+^), bile duct epithelial cells (CK19^+^), endothelial cells (CD31^+^), type 1 collagen, pan-keratin, E-cadherin, and nuclear DNA (Iridium 191/193); (b) viral markers HBsAg and HBcAg; and (c) immune markers including hematopoietic cells (CD45^+^) with various adaptive and innate immune subsets: CD4^+^ T cells (CD45^+^CD3^+^CD4^+^), CD8^+^ T cells (CD45^+^CD3^+^CD8^+^), B cells (CD45^+^CD20^+^), NK cells (CD45^+^ CD57^+^CD3^–^), and monocyte/macrophage subsets (CD45^+^CD68^+^, CD45^+^CD16^+^, CD45^+^CD14^+^, CD45^+^CD11b^+^). Additional markers for activation, memory, and/or function included HLA ABC, HLA DR, CD45RO, CD38, CD69, Ki67, granzyme B, perforin, PDL2, CD127, and CD107a. Supplemental Figure 1A shows representative staining characteristics for each antibody in the panel for a representative chronic hepatitis B subject. Efforts to stain for CD56, FoxP3, PD-1, and CD11c did not provide convincing results in our panel (data not shown).

### Tissue staining.

Tissue slides were processed as described previously (22), with image acquisition and downstream analyses as shown in Supplemental Figure 1B. Briefly, the FFPE liver slides (~4–5 μm in thickness) were deparaffinized in xylene and gradually rehydrated by sequential washes from 100% through 70% ethanol. Slides were then washed in PBS and transferred to Tris/EDTA (10 mM Tris, 1 mM EDTA, pH 9.2) buffer for antigen retrieval, which was performed in a decloaking chamber (Biocare Medical, DC2012) at 95°C for 30 minutes before being cooled at room temperature (RT) for 1 hour. Slides were then blocked in 3% BSA in PBS for 1 hour at RT and stained with ~100 μL of the antibody cocktail (Supplemental Table 2) overnight at 4°C in a humidified chamber. On the following day, slides were incubated for 30 minutes at RT with a cocktail of 1.25 μM Cell-ID Intercalator-Ir (Fluidigm, 2011192B) to stain for DNA and secondary antibodies. Slides were then washed 3 times in an excess (~40 mL) of ultrapure 18.2 milliQ water, and they were then air dried and acquired on the IMC (Fluidigm, Hyperion Imaging System).

### Image acquisition by CyTOF IMC.

As shown in Supplemental Figure 1B, ROIs in each slide/image were randomly selected based on bright-field images with inclusion of both portal and lobular regions. Image acquisition was carried out according to Fluidigm’s standard operating procedures, with a 200 Hz laser frequency and a 1 μm step increment. Typically, each 1 mm^2^ ROI required approximately 4 hours of acquisition. Given variations in the size and shape of available liver biopsy tissues, multiple adjacent ROIs were sequentially acquired and combined to construct approximately 1.8–2 mm^2^ total region per subject. As shown in Supplemental Table 3, the median acquired ROI was 1.9 mm^2^ overall, with marginal but significantly difference between chronic hepatitis B and NC subjects (median acquired ROI in mm^2^: IA 1.8 versus IT 1.8 versus NC 2.0, *P* = 0.006). Median percentage of PT per total acquired ROI was 5% overall, with greater percentage of PT region in IA compared with IT or NC subjects (IA 7.4% versus IT 2.5% versus NC 2.9%, *P* = 0.044). We used the percentage of portal area in total ROI acquired in each subject as a measure of fibrous expansion, a key element in Ishak staging for fibrosis (38, 39), based on these findings and apparent portal enrichment for type I collagen in IMC images.

### Image analysis and segmentation strategies.

Acquired images were reconstructed for all channels and multiple ROIs (Supplemental Figure 1B) and preprocessed in ImageJ (NIH) with a 3 × 3 pixel median filter to remove “salt-and-pepper” noise, followed by cell segmentation using CellProfiler (3.0.0) (47) to generate cell and PT masks (Supplemental Figure 1B and Supplemental Figure 2). The binary PT mask was manually defined in ImageJ, identifying PTs based on the detection of portal triad (CK19^+^ bile ducts, CD31^+^ endothelial cells, and portal vein) in addition to collagen in the absence of hepatocytes — typically with increased CD45^+^ cells in IA subjects. As for our cell segmentation or masking strategy to identify individual cells, because some large CD68^+^ and/or CD16^+^ cells were not associated with nuclear DNA (Supplemental Figure 2), we deployed 3 mutually exclusive cell masking strategies to identify all cell populations: (a) nuclear DNA^–^ cells that are positive for CD68 and/or CD16 (highlighted by cyan outlines in Supplemental Figure 2D); (b) nuclear DNA^+^ cells that are positive for CD68 and/or CD16 (highlighted by green outlines Supplemental Figure 2D); and (c) nuclear DNA^+^ cells that are negative for CD68 and CD16 (highlighted by violet outlines Supplemental Figure 2D). Multinucleated CD68^+^ and/or CD16^+^ cell aggregates were divided along presumed boundaries equidistant from their nuclei (red lines). To maximize cell separation and minimize overlap for immune cells that can be tightly clustered in portal regions, we defined 1 μm beyond the nucleus as the cellular area for nuclear DNA^+^CD68^–^CD16^–^ cells. CSV files were exported from CellProfiler with the following information on each segmented cell: expression levels for every marker included in the antibody panel and *x*/*y* coordinates to provide a 2-dimensional location within the acquired image. Cell channel values were normalized for each image by converting to *Z* scores as an internal standardization step to minimize variations between ROIs acquired over time. These results were further imported into R for downstream analysis with distinction for portal versus lobular location.

### Gating strategies for FCS files and for various adaptive and innate immune subsets.

CSV files exported from R were converted into flow cytometry standard (fcs) files, and ± cutoff values for each marker and sample were manually chosen in FlowJo based on biaxial plots using the CD45 channel for comparison. These cutoff values were then imported into R and used for gating, employing the flowCore and flowWorkspace packages, and subsequent statistical analyses (48, 49). The counts of total versus portal versus lobular events were examined, gating on CD45^+^ events for immune cells. For adaptive immune cells, CD45^+^ events were further gated for CD3^+^CD4^+^, CD3^+^CD8^+^, and CD20^+^ events as CD4^+^ T cells, CD8^+^ T cells, and CD20^+^ B cells, respectively. For innate immune cells, CD45^+^ events were further gated for CD68^+^, CD16^+^, CD14^+^, CD11b^+^, and CD57^+^ cells. Notably, expression patterns for CD68, CD16, and CD14 were highly colocalized with similar characteristics in quantitative and phenotype analyses (50). Therefore, we simplified our language and data presentation by using CD68 expression to define hepatic Kupffer cells, with a separate description of CD11b^+^ cells, which were also enriched for CD68 and HLA DR. We further defined CD45^+^CD3^–^CD57^+^ cells as a subset of mature NK cells that can accumulate with aging and may be altered in various viral infections, including chronic hepatitis B (40, 51). Gated immune subsets were then analyzed for phenotype marker expression. Results were normalized as hepatic cellular density in counts per mm^2^ ROI and as percentages for each cell subset of interest.

### Cluster analysis with PhenoGraph.

An initial cluster analysis with PhenoGraph (52) was then performed in R, with the inclusion of immune markers that defined our subsets (CD45, CD3, CD4, CD8, CD68, CD16, CD14, CD57, CD11b) and their phenotype (HLA DR, CD69, CD45RO, CD38, Ki67, granzyme B, perforin, CD107a), in addition to structural markers (HepPar1, CK19, HBsAg, HBcAg). In order to reduce bias in favor of samples with greater cell numbers, we used an equal number (total, 2697) of randomly selected cells from each sample, with a nearest neighbor setting of k = 100. This generated 19 clusters, 10 of which clustered together with enrichment for 1 or more immune markers (although not always enriched for CD45 expression) (Supplemental Figure 5, A and B). Cells in these 10 clusters were subjected to a second round of PhenoGraph (k = 100), again using an equal number (427 cells) of random cells per sample as input but including only immune markers without structural markers. The 13 resultant immune cell clusters were used to train a support vector machine (SVM) learning algorithm, which reclassified all cells, including those not previously input into either PhenoGraph analyses. In brief, the matrix of signal values and the derived cluster labels (10 immune-enriched clusters plus 1 cluster combining all other clusters) were provided to the R function “svm” in the package “e1071” (Meyer; x = signal matrix, y = cluster label) (53) to build a SVM model to convert signal patterns to cluster labels. The resulting model was then applied to a matrix containing the same signals for all cells using the R function “predict” to predict the cluster label for all cells. Both the “svm” and “predict” calls were made with the default parameters. We also evaluated other classifier approaches, including mean ± SD Gaussian models (MSD), kernel density function models (KDF), and R randomForest package, which performed less well compared with SVM. This yielded cluster assignments for the full data set for subsequent viSNE plots or heatmaps (Supplemental Figure 3B). We then selected 7 subclusters enriched for CD45 for further comparison with manually gated CD45^+^ immune subsets, as well as clinical parameters.

### Statistics.

Clinical or immune measures between 2 different groups were compared with the Mann-Whitney *U* test. Comparisons between IA, IT, and NC groups were made using the Kruskal-Wallis test (k = 3), followed by Mann-Whitney *U* test for further comparisons between 2 groups. Comparison of 2 related or matched samples (e.g., immune measures within the same cell subset or patients) were made with nonparametric matched-pair signed-rank test. Correlation between 2 immune measures was assessed using nonparametric Spearman’s correlation and the corresponding test. While *P* values below 0.05 were generally considered statistically significant, the threshold for statistical significance was further corrected for multiple comparisons, with an appreciation for its limitations (54).

### Study approval.

Study approval to obtain available liver tissues from NIDDK Central Repository and associated clinical parameters from the HBRN Data Coordinating Center for further analyses was obtained from the HBRN Steering Committee in addition to the IRB at the University of Pennsylvania and the Corporal Michael J. Crescenz VA Medical Center. The parent clinical study within the HBRN received approval by the IRB or equivalent committees for each of the centers participating in patient recruitment as previously described (9, 11, 29).

## Author contributions

KMC, DT, KBS, JS, DEK, ASL, WML, and KHK contributed to study design. DT, YJW, JS, and KMC conducted the experiments and acquired data. DT, KMC, JS, EEF, DEK, and ASW contributed to data analyses. DKHW, HLAJ, NAT, MK, ASW, KFM, PR, SCL, NRB, RKS, DTYL, TMB, MDF, EEF, KHK, and KMC provided the necessary reagents and resources for the study. KMC, DT, ASL, KS, DEK, KHK, JS, YJW, TMB, ASW, DKHW, HLAJ, NAT, MK, KM, PR, ACL, NRB, RKS, DTYL, MDF, EEF, and WML contributed to the manuscript writing.

## Supplementary Material

Supplemental data

## Figures and Tables

**Table 1:**
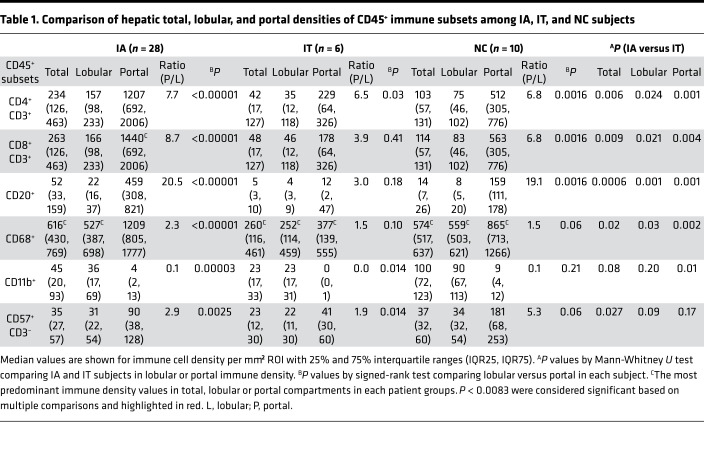
Comparison of hepatic total, lobular, and portal densities of CD45^+^ immune subsets among IA, IT, and NC subjects

## Version 1 02/23/2021

In-Press Preview

## Version 2 04/08/2021

Electronic publication
