# Supplementary material for: Highly multiplexed 2-dimensional imaging mass cytometry analysis of HBV-infected liver
Source: JCI Insight. 2021 Apr 8;6(7):e146883. doi: 10.1172/jci.insight.146883 (PMC8119221; doi:10.1172/jci.insight.146883)
Supplement: Supplemental data [file jciinsight-6-146883-s226.pdf]

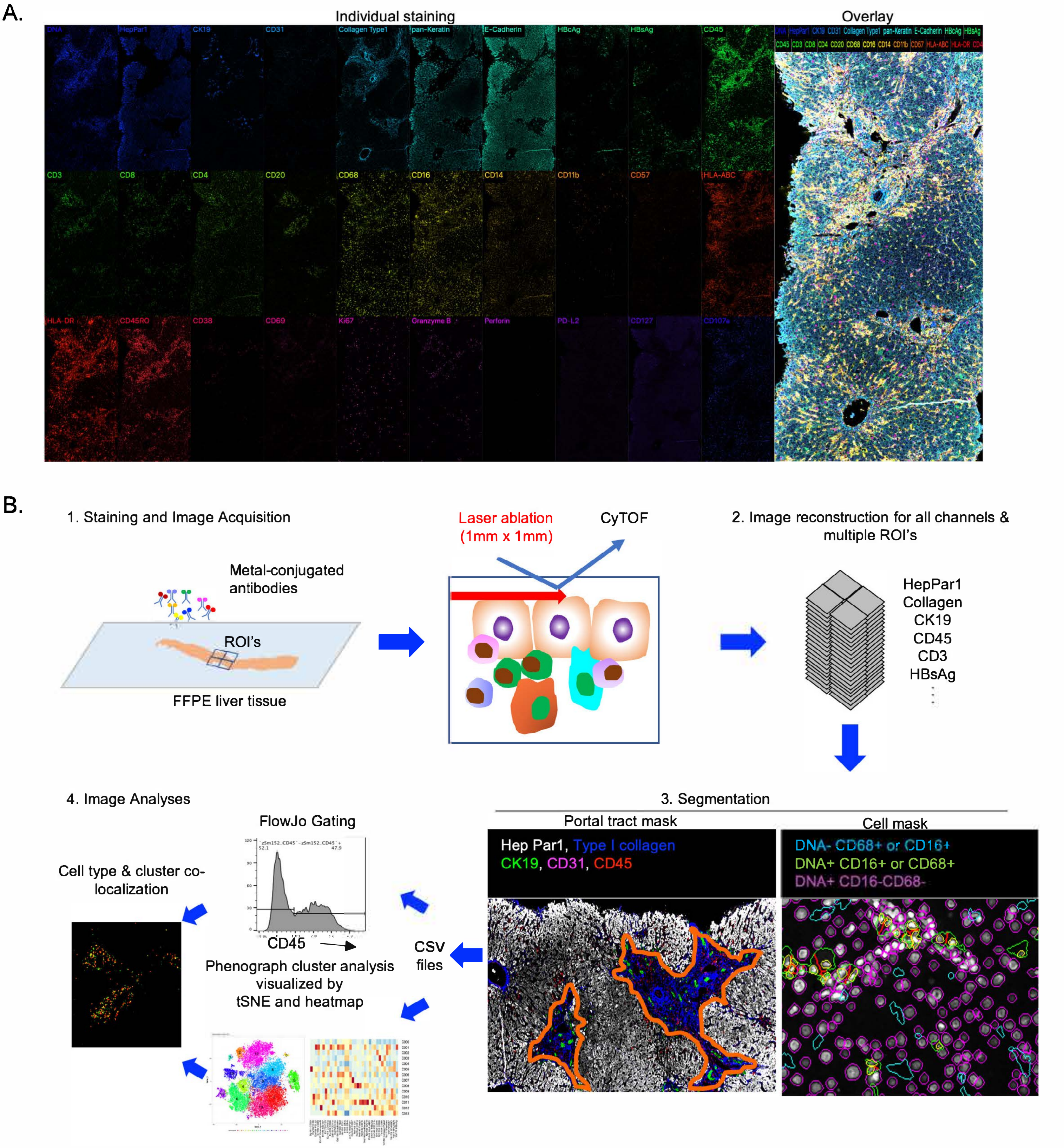

Supplementary Figure S1. Antibody panel, Image processing and Analytic platform. A. Staining characteristics for antibodies in the panel shown individually and as a combined overlay (IA-A13). B. Image processing and analytic platform: 1) Staining and acquisition of formalin-fixed paraffin embedded (FFPE) liver tissue on slides with a cocktail of metal-conjugated antibodies, followed by laser-ablation of randomly selected regions of interest (ROI's) with CyTOF. 2. Image reconstruction for all channels and multiple ROI's for all markers included in the antibody panel. 3. Segmentation of acquired and reconstructed images by (left image) portal tract mask (orange outlines) manually gated based on the presence of CK19+ bile ducts, CD31+ vessels and collagen without HepPar1+ hepatocytes; and (right image) cell mask based on nuclear DNA, CD68 and/or CD16 expression (see also Supplementary Figure S2). 4. Image analyses of resulting data by FlowJo and RPhenograph cluster analysis (visualized by tSNE plots and heatmaps), with further co-localization of cell types and clusters.

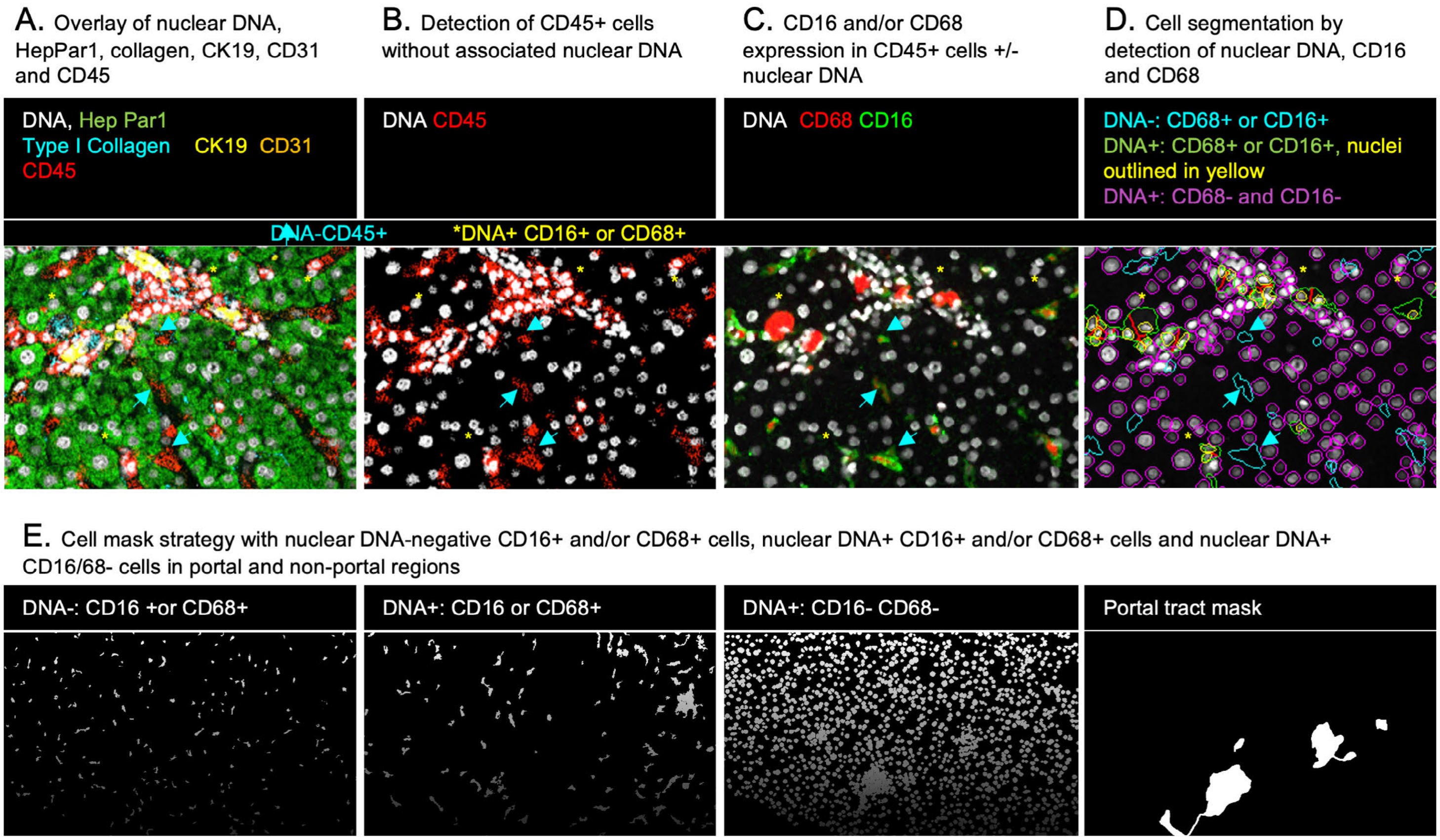

Supplementary Figure S2. Cell segmentation strategy. Cell segmentation strategy using masks for cells and portal tracts is shown, based on detection of CD68+ and/or CD16+ cells with and without associated nuclear DNA. A. Overlay of nuclear DNA (gray), HepPar1 (green), type 1 collagen (cyan), CK19 (yellow), CD31 (orange) and CD45 (red). B. Detection of CD45+ cells without associated nuclear DNA in select overlay of nuclear DNA (gray) and CD45 (red). Cyan arrows identify red CD45+ staining without associated with nuclear DNA, whereas yellow asterisks identify red CD45+ cells associated with nuclear DNA. C. CD16 and/or CD68 expression in CD45+ cells with and without nuclear DNA in select overlay of nuclear DNA (gray), CD68 (red) and CD16 (green). Cyan arrows highlight select CD68+ and/or CD16+ cells without associated nuclear DNA. Yellow asterisks highlight select CD68+ and/or CD16+ cells with associated nuclear DNA. D. Cell segmentation by detection of nuclear DNA, CD16 and CD68 provides outlines for various cell types that are mutually exclusive: Cyan outline for DNA-negative CD68+ or CD16+ cells; Green outline for DNA-positive CD68+ and/or CD16+ cells with associated nuclei outlined in yellow; Violet outline for remaining CD68-/CD16- nuclear DNA+ with the border at 1 micron beyond the outer edge of nuclear DNA staining. Green outlines containing multiple nuclei were further subdivided with equal distance between the nuclei and the subdividing line (shown in red line). E. Cell mask strategy with nuclear DNA-negative CD16+ and/or CD68+ cells, nuclear DNA+ CD16+ and/or CD68+ cells and nuclear DNA+ CD16/68- cells in portal and non-portal regions

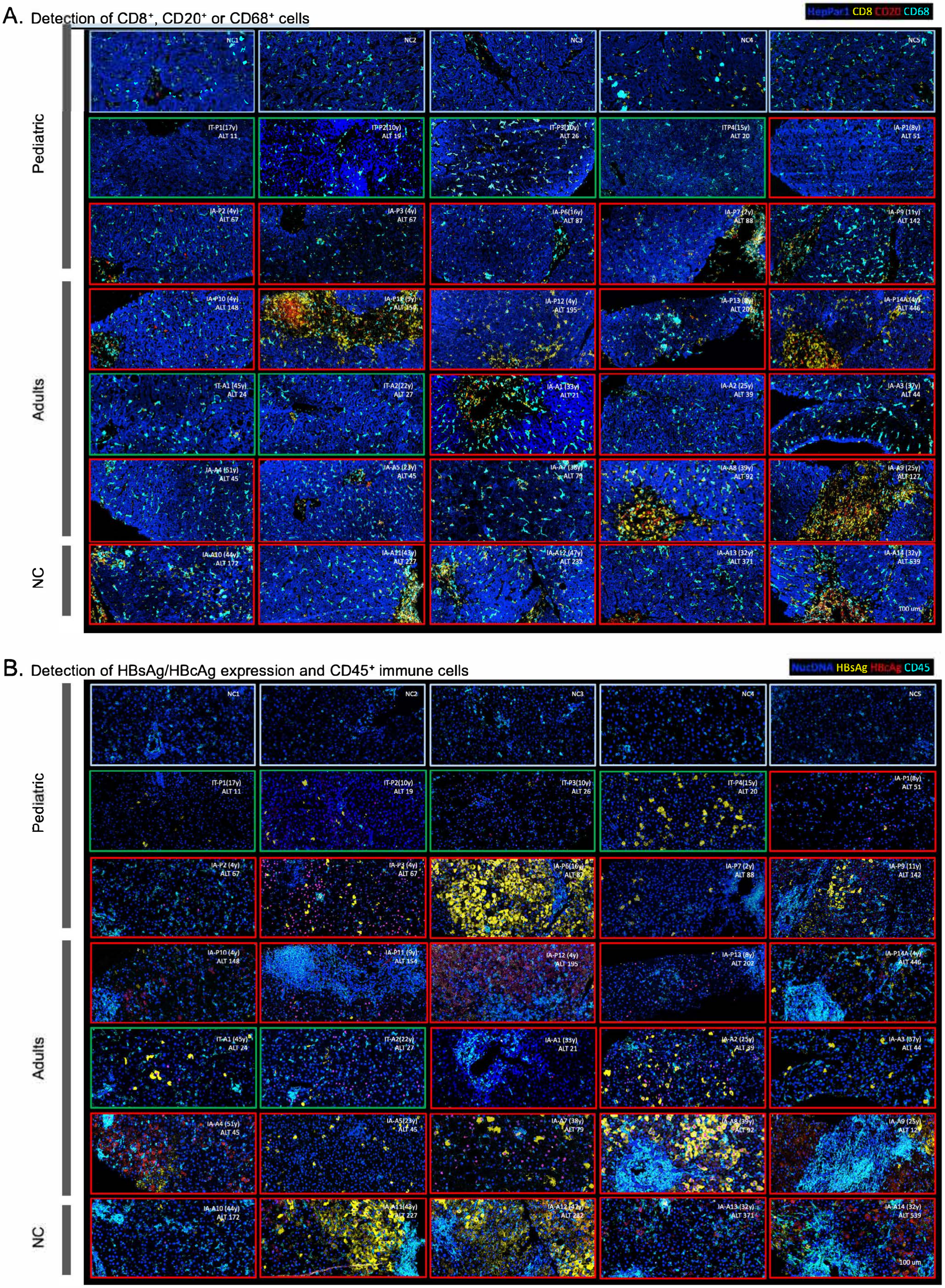

Supplementary Figure S3. Detection of immune cells and HBsAg/HBcAg expression in the liver of children and adults with HBeAg+ immune tolerant (IT) and immune active (IA) chronic hepatitis B. A. Detection of $CD8^{+}$, $CD20^{+}$ or $CD68^{+}$ cells. B. Detection of $CD45^{+}$ immune cells and hepatocytes with HBsAg/HBcAg expression. The panels include representative views within acquired ROI's from 15 pediatric (4 IT, 11 IA), 15 adult (3 IT, 12 IA) and 5 non-infected NC subjects. Images are outlined in white for NC, green for IT and red for IA subjects, and labeled with subject ID (age in years), serum ALT in U/L and HBV DNA in log U/ml.

A. P-values associated with Spearman correlation coefficients in Figure 4C.

	Total						Lobular						Portal					
	$CD4^{+}CD3^{+}$	$CD8^{+}CD3^{+}$	$CD20^{+}$	$CD68^{+}$	$CD11b^{+}$	$CD57^{+}CD3^{-}$	$CD4^{+}CD3^{+}$	$CD8^{+}CD3^{+}$	$CD20^{+}$	$CD68^{+}$	$CD11b^{+}$	$CD57^{+}CD3^{-}$	$CD4^{+}CD3^{+}$	$CD8^{+}CD3^{+}$	$CD20^{+}$	$CD68^{+}$	$CD11b^{+}$	$CD57^{+}CD3^{-}$
	p-value for spearman correiation with serum ALT																	
%HLA ABC+	9.E-01	3.E-01	5.E-01	4.E-01	1.E-02	8.E-01	6.E-01	2.E-01	1.E+00	1.E-01	9.E-03	7.E-01	1.E-01	3.E-01	9.E-01	7.E-01	6.E-02	4.E-01
%HLA DR+	6.E-03	1.E-03	3.E-01	1.E-03	7.E-01	3.E-01	1.E-02	6.E-03	3.E-01	9.E-04	5.E-01	4.E-01	4.E-01	7.E-01	2.E-01	2.E-01	2.E-01	7.E-01
%CD45RO+	2.E-03	4.E-03	6.E-03	2.E-03	1.E+00	3.E-04	2.E-02	9.E-03	8.E-02	8.E-03	4.E-01	1.E-03	9.E-01	2.E-01	2.E-01	2.E-02	2.E-01	6.E-04
%CD38+	5.E-02	2.E-03	5.E-01	7.E-03	6.E-02	6.E-02	3.E-02	2.E-02	4.E-01	3.E-02	4.E-02	7.E-02	5.E-02	3.E-03	1.E-01	7.E-03	9.E-02	1.E+00
%CD69+	9.E-01	1.E+00	7.E-01	5.E-01	1.E-01	9.E-01	8.E-01	8.E-01	9.E-01	8.E-01	3.E-01	6.E-01	1.E+00	5.E-01	9.E-01	7.E-01	3.E-01	1.E+00
%Ki67+	3.E-01	6.E-01	2.E-01	5.E-01	9.E-01	1.E+00	7.E-01	9.E-01	5.E-01	9.E-01	8.E-01	9.E-01	4.E-01	2.E-01	5.E-01	3.E-01	2.E-01	9.E-01
%Granzyme	4.E-01	4.E-01	6.E-01	3.E-01	1.E-01	9.E-01	7.E-01	6.E-01	9.E-01	8.E-03	2.E-02	7.E-01	5.E-02	1.E-02	5.E-02	7.E-02	2.E-01	2.E-01
%Perforin+	4.E-01	1.E+00	8.E-01	6.E-01	1.E-01	2.E-01	3.E-01	8.E-01	2.E-01	5.E-01	5.E-02	2.E-01	4.E-01	3.E-01	4.E-01	4.E-01	6.E-02	3.E-01
	p-value for spearman correiation with serum HBV DNA																	
%HLA ABC+	4.E-02	2.E-01	2.E-01	1.E-01	4.E-01	2.E-01	9.E-03	3.E-01	1.E-01	3.E-01	4.E-01	1.E-01	3.E-01	2.E-02	5.E-01	3.E-01	8.E-02	3.E-01
%HLA DR+	1.E+00	7.E-01	9.E-01	1.E-01	8.E-01	7.E-01	9.E-01	1.E+00	7.E-01	1.E-01	7.E-01	5.E-01	9.E-01	6.E-01	8.E-01	6.E-01	3.E-01	5.E-01
%CD45RO+	9.E-01	8.E-01	7.E-01	5.E-01	5.E-01	5.E-01	5.E-01	9.E-01	4.E-01	7.E-01	3.E-01	9.E-01	4.E-01	8.E-01	6.E-01	6.E-01	6.E-01	5.E-01
%CD38+	7.E-01	4.E-01	4.E-01	1.E-02	5.E-02	3.E-01	9.E-01	2.E-01	9.E-01	2.E-02	3.E-02	2.E-01	9.E-01	7.E-01	3.E-01	2.E-01	9.E-01	3.E-01
%CD69+	5.E-01	7.E-01	4.E-01	1.E+00	8.E-01	6.E-01	4.E-01	7.E-01	8.E-01	1.E+00	1.E+00	1.E+00	8.E-01	7.E-01	9.E-01	4.E-01	5.E-01	6.E-01
%Ki67+	8.E-01	6.E-01	9.E-01	4.E-01	6.E-01	5.E-01	5.E-01	6.E-01	7.E-01	4.E-01	5.E-01	7.E-01	7.E-01	5.E-01	7.E-01	4.E-01	3.E-01	2.E-01
%Granzyme	3.E-01	5.E-02	6.E-01	9.E-02	8.E-01	5.E-01	4.E-01	9.E-02	5.E-01	3.E-01	7.E-01	6.E-01	1.E+00	1.E+00	3.E-01	4.E-01	3.E-01	2.E-01
%Perforin+	3.E-01	3.E-02	5.E-01	1.E-02	9.E-02	2.E-01	2.E-01	1.E-02	3.E-01	1.E-02	2.E-02	1.E-01	1.E-01	4.E-01	2.E-01	1.E-01	6.E-01	6.E-01
	p-value for spearman correiation with age																	
%HLA ABC+	6.E-01	8.E-01	7.E-01	1.E+00	9.E-01	3.E-01	4.E-01	9.E-01	8.E-01	1.E+00	9.E-01	3.E-01	9.E-01	1.E+00	8.E-01	8.E-01	9.E-01	5.E-01
%HLA DR+	6.E-01	8.E-01	8.E-01	5.E-02	6.E-01	3.E-01	6.E-01	9.E-01	7.E-01	6.E-02	5.E-01	2.E-01	7.E-01	7.E-01	7.E-01	2.E-01	8.E-01	4.E-01
%CD45RO+	2.E-01	8.E-02	7.E-01	1.E+00	4.E-01	4.E-01	4.E-02	2.E-02	7.E-01	7.E-01	4.E-01	4.E-01	7.E-02	3.E-02	4.E-01	5.E-01	8.E-01	6.E-01
%CD38+	4.E-02	5.E-01	8.E-03	9.E-01	3.E-01	8.E-01	2.E-01	4.E-01	1.E-01	1.E+00	4.E-01	6.E-01	6.E-03	9.E-02	5.E-02	4.E-02	5.E-01	1.E-02
%CD69+	3.E-02	1.E-01	2.E-01	6.E-01	9.E-01	7.E-01	6.E-02	2.E-01	2.E-01	8.E-01	6.E-01	7.E-01	2.E-02	3.E-02	4.E-01	8.E-02	2.E-01	2.E-01
%Ki67+	8.E-01	5.E-01	9.E-01	8.E-01	6.E-01	6.E-01	6.E-01	8.E-01	5.E-01	6.E-01	6.E-01	9.E-01	5.E-01	8.E-01	7.E-01	4.E-01	2.E-01	9.E-01
%Granzyme	1.E+00	9.E-01	3.E-01	3.E-01	4.E-01	4.E-01	6.E-01	8.E-01	3.E-01	5.E-01	5.E-01	4.E-01	3.E-01	8.E-01	9.E-01	2.E-01	8.E-01	1.E+00
%Perforin+	6.E-01	9.E-01	5.E-01	4.E-01	8.E-01	4.E-01	7.E-01	4.E-01	6.E-01	2.E-01	8.E-01	5.E-01	8.E-02	9.E-02	3.E-01	3.E-01	2.E-02	4.E-01

	Total						Lobular						Portal					
	$CD4^{+}CD3^{+}$	$CD8^{+}CD3^{+}$	$CD20^{+}$	$CD68^{+}$	$CD11b^{+}$	$CD57^{+}CD3^{-}$	$CD4^{+}CD3^{+}$	$CD8^{+}CD3^{+}$	$CD20^{+}$	$CD68^{+}$	$CD11b^{+}$	$CD57^{+}CD3^{-}$	$CD4^{+}CD3^{+}$	$CD8^{+}CD3^{+}$	$CD20^{+}$	$CD68^{+}$	$CD11b^{+}$	$CD57^{+}CD3^{-}$
	p-value for spearman correiation with Ishak lobular inflammatory score																	
%HLA ABC+	5.E-01	1.E-01	7.E-01	3.E-01	7.E-03	7.E-01	4.E-01	2.E-01	1.E+00	1.E-01	4.E-03	6.E-01	6.E-01	8.E-02	8.E-01	8.E-01	1.E-01	3.E-01
%HLA DR+	4.E-03	1.E-03	8.E-02	3.E-02	8.E-01	6.E-03	4.E-04	1.E-03	1.E-01	1.E-02	6.E-01	1.E-02	7.E-01	5.E-01	5.E-01	9.E-02	1.E+00	1.E-01
%CD45RO+	3.E-02	5.E-02	6.E-02	3.E-02	6.E-01	7.E-03	3.E-02	3.E-02	2.E-01	4.E-02	2.E-01	3.E-02	1.E+00	5.E-01	9.E-01	6.E-02	9.E-01	1.E-03
%CD38+	3.E-03	2.E-02	4.E-01	3.E-02	9.E-02	2.E-01	2.E-02	6.E-02	5.E-01	1.E-01	4.E-02	1.E-01	2.E-03	4.E-04	1.E-01	2.E-04	3.E-01	8.E-01
%CD69+	3.E-01	9.E-01	9.E-01	5.E-01	2.E-01	2.E-01	5.E-01	7.E-01	8.E-01	1.E+00	4.E-01	6.E-01	7.E-01	4.E-01	1.E+00	2.E-01	3.E-01	8.E-01
%Ki67+	1.E-01	9.E-01	4.E-01	9.E-01	1.E+00	8.E-01	5.E-01	7.E-01	6.E-01	4.E-01	1.E+00	9.E-01	3.E-01	3.E-01	5.E-01	3.E-01	2.E-01	9.E-01
%Granzyme	8.E-01	6.E-01	8.E-01	8.E-03	8.E-03	6.E-01	7.E-01	5.E-02	6.E-01	4.E-05	1.E-03	8.E-01	2.E-01	3.E-02	3.E-01	6.E-01	8.E-01	9.E-02
%Perforin+	6.E-01	5.E-01	8.E-01	6.E-01	2.E-01	5.E-01	4.E-01	6.E-01	5.E-01	4.E-01	8.E-02	6.E-01	4.E-01	2.E-01	8.E-01	4.E-01	4.E-02	2.E-01
	p-value for spearman correiation with Ishak periportal inflammatory score																	
%HLA ABC+	6.E-01	1.E-01	6.E-01	3.E-02	9.E-04	6.E-01	5.E-01	7.E-02	1.E+00	5.E-03	1.E-03	1.E+00	2.E-01	5.E-02	1.E+00	9.E-01	7.E-02	2.E-01
%HLA DR+	2.E-03	9.E-04	2.E-02	7.E-04	7.E-01	1.E-01	2.E-02	1.E-02	3.E-02	7.E-04	1.E+00	2.E-01	5.E-01	1.E-01	2.E-01	3.E-01	7.E-01	5.E-01
%CD45RO+	1.E-03	3.E-03	7.E-03	6.E-04	9.E-01	3.E-04	4.E-03	4.E-03	4.E-02	6.E-03	4.E-01	1.E-03	6.E-01	3.E-01	5.E-01	1.E-02	5.E-01	1.E-03
%CD38+	7.E-02	5.E-02	8.E-01	4.E-03	7.E-02	1.E-01	2.E-01	2.E-01	1.E+00	4.E-02	6.E-02	1.E-01	8.E-02	7.E-04	1.E-01	5.E-03	7.E-02	1.E+00
%CD69+	6.E-01	3.E-01	4.E-01	4.E-01	4.E-01	6.E-02	4.E-01	2.E-01	4.E-01	1.E-01	5.E-01	3.E-01	4.E-01	6.E-01	6.E-01	8.E-01	1.E-01	1.E-01
%Ki67+	8.E-01	6.E-01	2.E-01	8.E-01	9.E-01	6.E-01	6.E-01	3.E-01	4.E-01	1.E-01	1.E+00	5.E-01	1.E+00	6.E-01	3.E-01	4.E-01	1.E-01	1.E+00
%Granzyme	7.E-01	6.E-01	9.E-01	1.E-01	8.E-02	6.E-01	5.E-01	6.E-02	9.E-01	2.E-03	6.E-03	5.E-01	1.E-02	6.E-03	5.E-02	2.E-01	6.E-01	1.E-01
%Perforin+	9.E-01	3.E-01	1.E+00	5.E-01	4.E-01	3.E-01	6.E-01	4.E-01	7.E-01	4.E-01	1.E-01	3.E-01	5.E-01	8.E-02	1.E-02	6.E-01	1.E-02	2.E-01
	p-value for spearman correiation with Ishak fibrosis score																	
%HLA ABC+	5.E-01	3.E-01	7.E-01	6.E-02	2.E-04	6.E-01	7.E-01	2.E-01	5.E-01	2.E-02	4.E-04	9.E-01	8.E-01	1.E-01	9.E-01	8.E-01	5.E-02	1.E-01
%HLA DR+	1.E-05	3.E-07	7.E-03	6.E-04	4.E-01	1.E-04	2.E-05	4.E-06	2.E-02	3.E-04	6.E-01	1.E-03	8.E-01	2.E-01	1.E-01	7.E-01	3.E-01	2.E-02
%CD45RO+	1.E-02	8.E-03	9.E-04	2.E-03	8.E-01	7.E-04	2.E-02	6.E-03	1.E-02	1.E-02	4.E-01	7.E-03	9.E-01	5.E-01	3.E-01	2.E-02	5.E-01	2.E-03
%CD38+	8.E-02	4.E-02	8.E-01	3.E-02	4.E-02	7.E-01	4.E-02	2.E-02	7.E-01	1.E-01	3.E-02	5.E-01	2.E-02	1.E-02	1.E-01	4.E-04	7.E-02	4.E-01
%CD69+	2.E-01	5.E-01	6.E-01	3.E-01	1.E-02	3.E-01	3.E-01	6.E-01	8.E-01	7.E-01	1.E-02	9.E-01	2.E-01	2.E-01	2.E-01	9.E-02	3.E-02	8.E-02
%Ki67+	2.E-01	9.E-01	8.E-01	5.E-01	4.E-01	4.E-01	7.E-01	6.E-01	7.E-01	5.E-01	5.E-01	3.E-01	1.E-01	4.E-01	1.E-01	2.E-01	1.E-01	6.E-01
%Granzyme	2.E-02	2.E-01	9.E-02	5.E-01	3.E-01	8.E-02	4.E-01	7.E-01	1.E-01	2.E-02	3.E-02	2.E-01	2.E-03	1.E-04	3.E-04	4.E-02	5.E-01	7.E-02
%Perforin+	5.E-01	6.E-01	8.E-01	8.E-01	2.E-01	9.E-01	1.E-01	1.E+00	2.E-01	7.E-01	4.E-02	7.E-01	1.E-01	1.E-01	6.E-02	8.E-02	7.E-03	3.E-01

B. Dot plots comparing IA and IT subjects for %HLA DR+ or %CD45RO+ cells within each immune compartments.

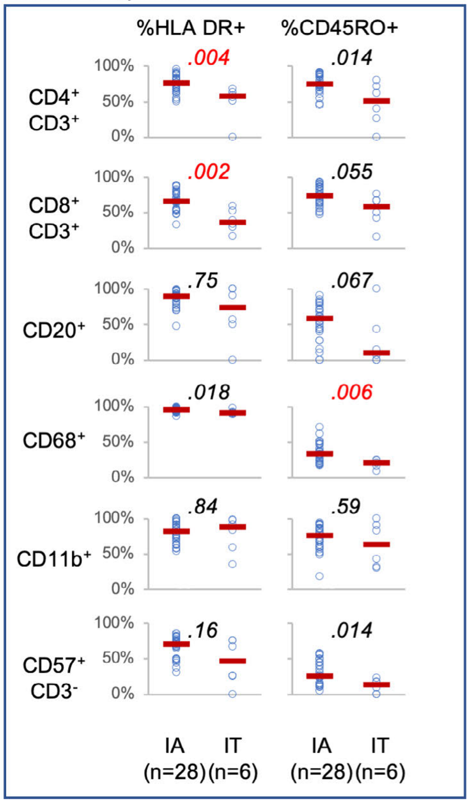

C. Scatter plots correlating %HLA DR+ or %CD45RO+ cells within each immune compartment with ALT, HBV DNA and fibrosis scores.

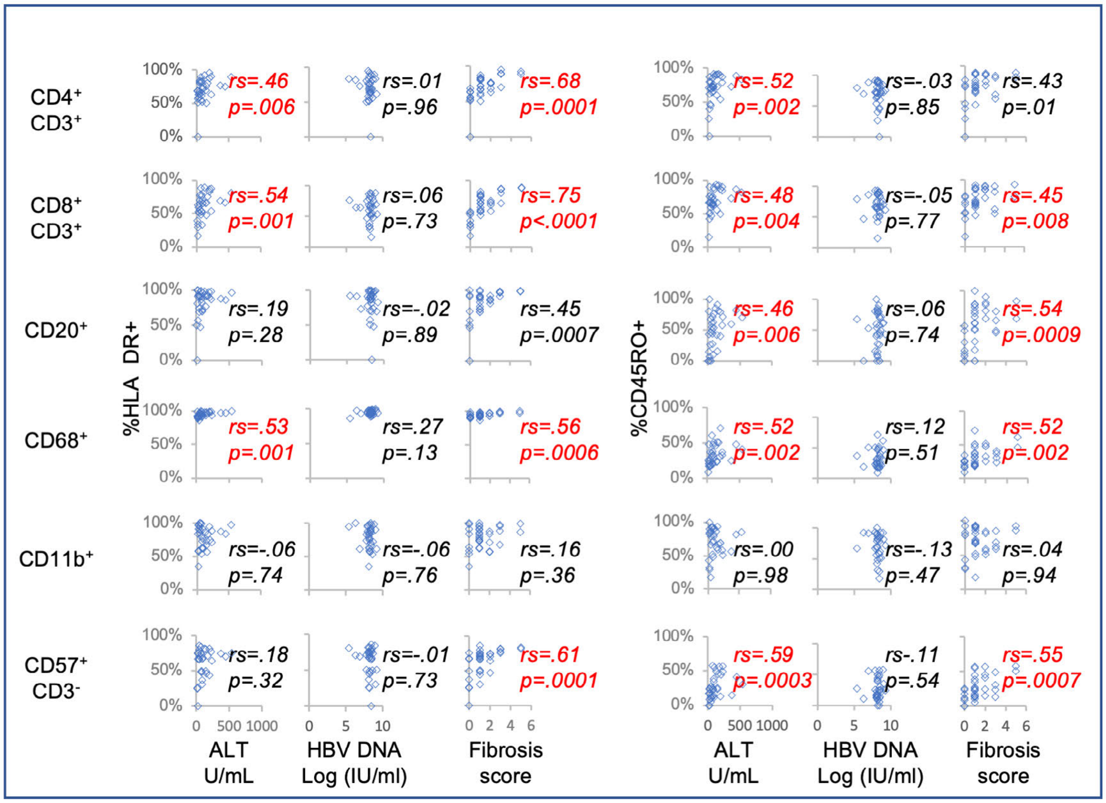

Supplementary Figure S4. Correlations between adaptive and innate immune phenotype and clinical, virological and histological markers. A. P-values for Spearman correlation coefficients in Figures 4C/D. P-values <0.00625 highlighted in red font with pink shade. B. Dot plots comparing IA and IT subjects for %HLA DR+ or %CD45RO+ cells within each immune compartment, with horizontal red lines indicating median values and associated p-values shown via italicized numbers based on Mann-Whitney U. P-values <.00625 highlighted in red font. C. Scatter plots correlating %HLA DR+ or %CD45RO+ cells within each immune compartment with ALT, HBV DNA and fibrosis scores. Spearman correlation (rs) and associated p-values—highlighted in red font for p-values <0.00625.

D. Broad correlations between activation and effector phenotypes of hepatic adaptive and innate immune subsets shown as a heatmap

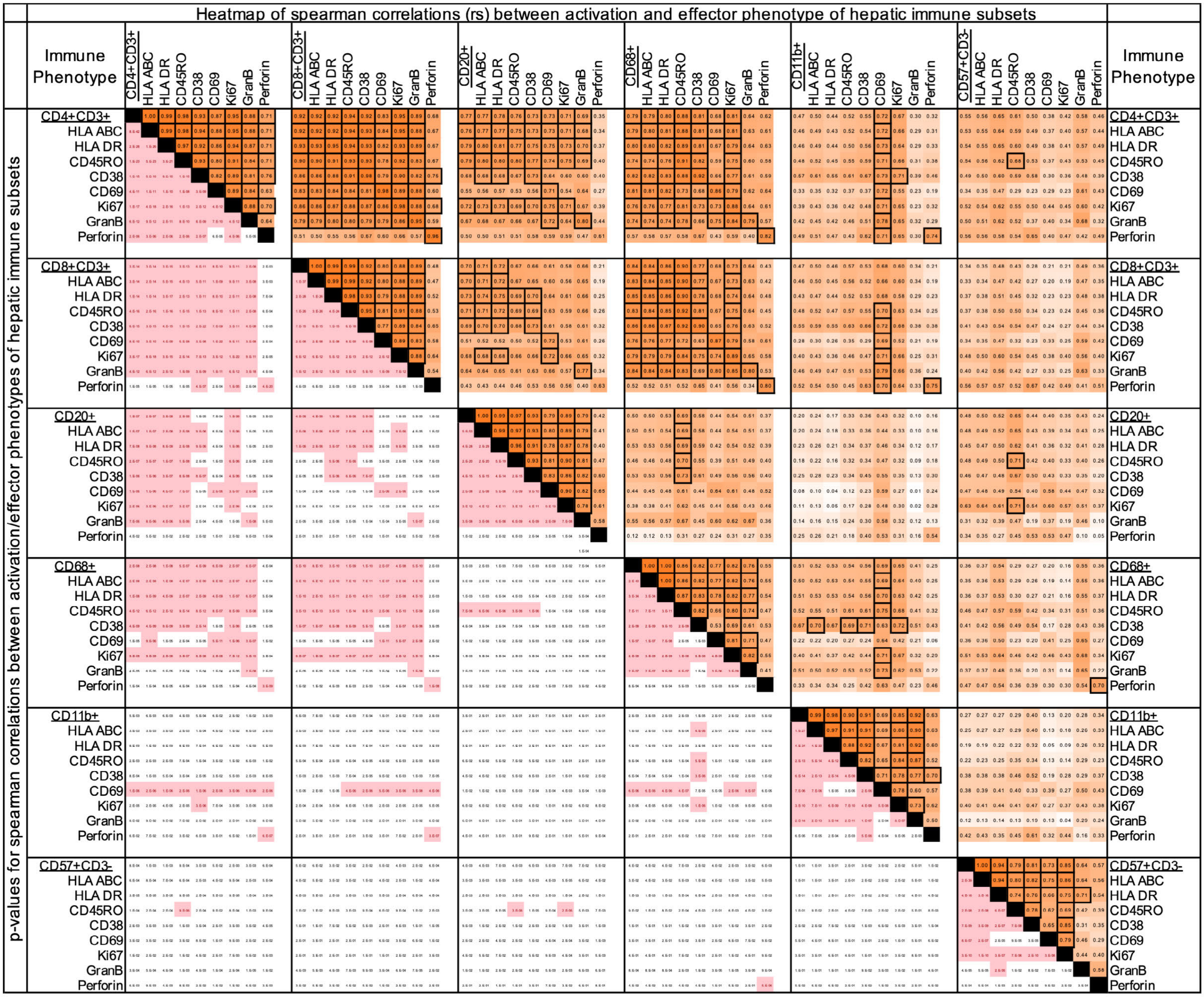

Supplementary Figure S4D. Broad correlations between activation and effector phenotypes of hepatic adaptive and innate immune subsets shown as a heatmap of Spearman Correlation Coefficients (rs) on the upper right half (with positive correlations defined by the extent of orange shade) and associated p-values on the lower left half in a mirror image. Correlations with p-values < 0.00000965 were considered significant and highlighted by dark border, with p-values indicated via pink shade. As shown, numerous correlations reached statistical significance for activation and effector phenotype marker expression: 1) within each immune subsets; 2) between adaptive immune subsets (e.g. between CD4+CD3+ and CD8+CD3+ cells, CD4+ and CD20+ cells, CD8+ and CD20+ cells); 3) between adaptive and innate immune subsets (e.g. between CD4+CD3+ and CD68+ cells, CD8+CD3+ and CD68+ cells, CD20+ and CD68+ cells, CD11b+ and CD4+CD3+ cells, CD11b+ and CD8+CD4+ cells). Correlations were less broad for CD11b+ and CD57+CD3- cells, although CD69 expression in CD11b+ cells correlated with activation phenotype of CD4+CD3+, CD8+CD3+ and CD68+ cells. Collectively, these findings show close interactions between hepatic immune subsets with significant correlations in hepatic density as well as activation and effector phenotypes.

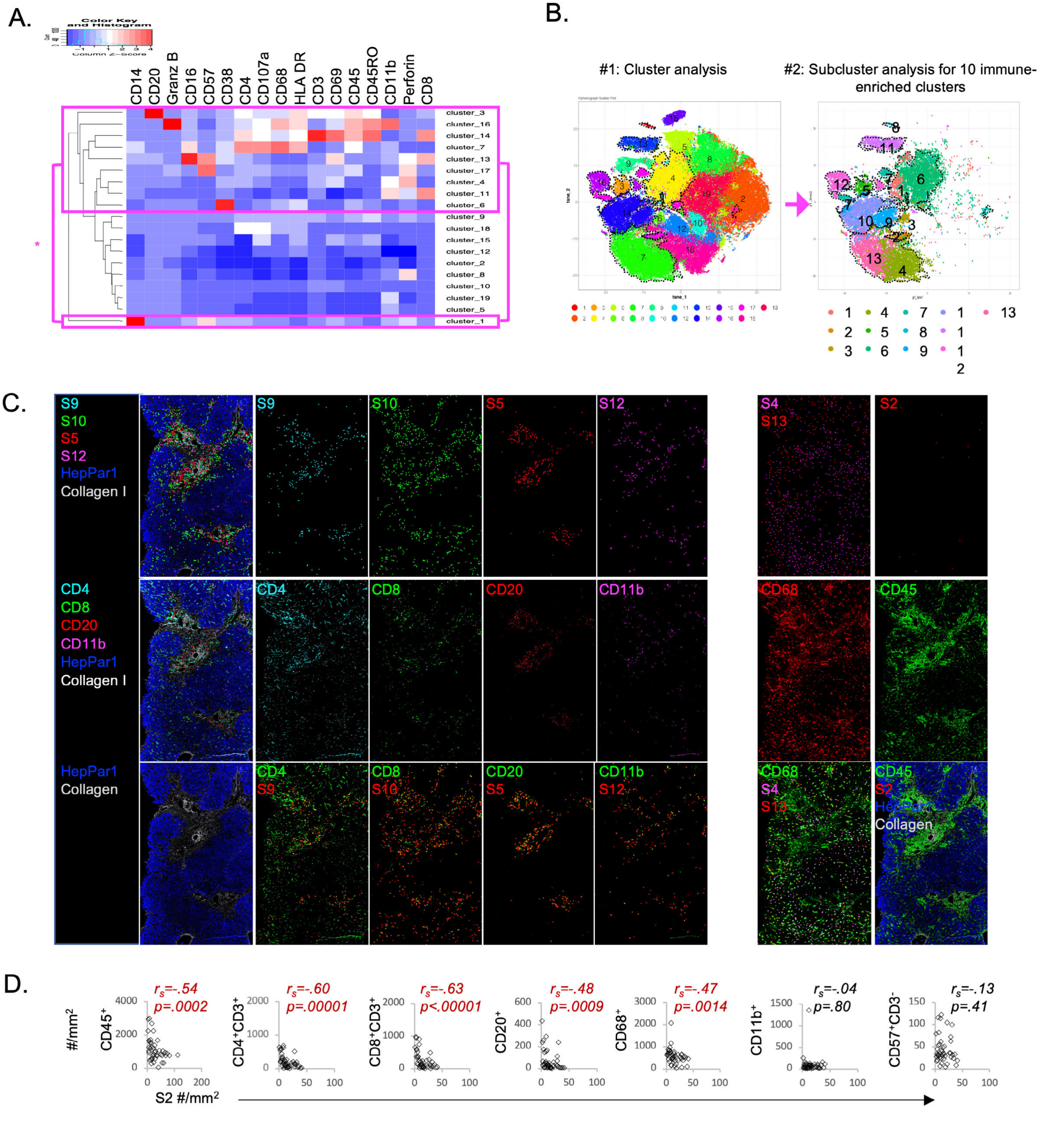

Supplementary Figure S5. Adaptive and innate immune subsets defined by PhenoGraph analysis. A. Initial PhenoGraph analysis with hierarchical clustering defined 10 clusters (top 9: clusters 3, 16, 14, 7, 13, 17, 4, 11 and 6 in addition to bottom cluster 1) among 19 identified based on enrichment for one or more immune markers among the hierarchical clusters. B. tSNE plots of PhenoGraph analyses. tSNE plot of the initial cluster analysis (#1) is shown on the left, with the tSNE plot on the right showing the localization of subsequently identified subclusters (#2) from 10 immune-enriched clusters. The second tSNE plot shows the subdivisions of cluster 7 (into subclusters 4 and 13), cluster 14 (into subclusters 9 and 10) and cluster 17 (into subcluster 2, 7). C. Distribution of events in immune subclusters S5, 9, 10, 12, 13 and 2 and their overlays with corresponding immune subsets. HepPar1+ (blue) and collagen (gray) provide the hepatic architecture in addition to immune markers CD20, CD4, CD8, CD11b, CD68 and CD45. S2+ cells were CD45+ without other corresponding markers in our panel. D. Scatter plots of cell densities/mm^2 of ROI for S2 subcluster and various adaptive and innate immune subsets. Significant inverse correlations are shown by Spearman Correlation (highlighted in red font for p-values <.00714).

Supplementary Table S1.

		Demographic Parameters			Clinical and Virological Parameters						Ishak Inflammation			Ishak Fibrosis	
**HBV Groups**	**ID**	**F/M**	**race**	**Age years**	**ALT U/L**	**Platelets x10^3/ml**	**Albumin (g/dL)**	**INR**	**HBV DNA log IU/ml**	**HBV Genotype**	**Lobular**	**Portal**	**peri-portal**	**Overall**	**Sinusoidal**
**IT-Ped (n=4)**	**IT-P1**	F	Asian	17	11	180	4.6	1.1	8.1	B	1	1	1	0	0
	**IT-P2**	F	Asian	10	19	296	4.6	1.0	8.2	B	1	1	1	0	0
	**IT-P3**	F	Asian	15	20	174	5.2	1.1	8.4	B	1	1	1	0	0
	**IT-P4**	F	Asian	10	26	264	4.3	1.0	8.0	C	1	1	1	1	0
**IT-Adult (n=2)**	**IT-A1**	M	Asian	45	24	310	4.3	NA	8.1	NA	1	0	0	0	1
	**IT-A2**	M	Asian	22	27	189	3.9	1.0	8.2	B	2	1	1	0	0
**IA-Ped (n=14)**	**IA-P1**	F	Asian	8	51	256	4.3	1.1	6.3	NA	2	2	2	1	0
	**IA-P2**	F	Asian	4	67	335	3.7	1.0	8.1	B	1	2	1	1	0
	**IA-P3**	M	White	5	67	279	NA	1.0	8.6	D	1	0	0	0	0
	**IA-P4**	F	Asian	9	69	180	1.1	1.0	8.9	C	2	2	3	5	0
	**IA-P5**	F	Asian	4	76	231	4.1	0.9	8.7	C	2	2	1	1	0
	**IA-P6**	F	Asian	16	87	310	4.3	1.0	8.1	B	1	1	1	0	0
	**IA-P7**	F	Asian	2	88	432	5.1	1.1	9.0	C	1	1	1	1	0
	**IA-P8**	F	Asian	15	99	181	4.4	1.0	6.9	B	3	2	2	1	0
	**IA-P9**	F	Asian	11	142	264	4.2	1.0	8.5	B	4	2	2	3	1
	**IA-P10**	M	Asian	4	148	235	4.1	1.0	7.8	C	2	2	2	1	0
	**IA-P11**	F	Asian	9	154	211	4.3	1.1	8.3	D	3	3	4	2	2
	**IA-P12**	F	Asian	4	195	242	4.1	1.1	8.0	B	4	2	3	3	1
	**IA-P13**	M	Black	8	202	219	4.1	1.1	8.2	E	2	1	2	1	0
	**IA-P14**	M	Mixed	4	**446**	269	4.4	1.1	8.2	B	3	2	2	2	1
**IA-Adult (n=14)**	**IA-A1**	M	Asian	33	21	164	4.0	1.0	8.3	C	2	2	2	2	1
	**IA-A2**	F	Asian	25	39	239	4.2	1.1	8.4	C	1	0	0	0	1
	**IA-A3**	M	Asian	37	44	NA	4.5	NA	8.4	A	2	1	0	1	0
	**IA-A4**	F	Asian	51	45	226	4.1	NA	5.4	C	2	2	1	1	0
	**IA-A5**	M	Asian	23	45	214	3.9	1.1	8.5	C	2	1	1	1	0
	**IA-A6**	M	Asian	23	79	244	4.6	1.0	8.2	B	2	1	1	1	0
	**IA-A7**	F	Asian	38	79	370	4.2	1.1	9.2	D	2	2	2	1	0
	**IA-A8**	M	Asian	39	92	190	4.0	1.1	8.2	D	2	2	2	1	0
	**IA-A9**	M	Asian	25	127	193	4.2	1.0	8.2	C	2	2	3	5	0
	**IA-A10**	F	Asian	44	172	188	4.0	0.9	7.8	B	3	1	2	1	0
	**IA-A11**	M	Asian	43	227	333	3.9	1.0	8.4	C	3	3	4	3	0
	**IA-A12**	M	Asian	47	232	248	3.7	0.9	8.4	B	3	2	3	2	0
	**IA-A13**	M	Asian	32	**371**	150	4	0.9	8	C	3	1	3	2	0
	**IA-A14**	M	Asian	32	**539**	195	4.3	1.2	8.5	B	4	1	3	3	0
		**F/M**	**#Asians**	**Median values**						**A/B/C/D/E**	**Median values**				
**All IT**		4/2	6/6	16	22	227	4.5	1.0	8.2	0/4/1/0/0	1	1	1	0	0
**IT-Pediatric**		4/0	4/4	12	20	222	4.6	1.1	8.1	0/3/1/0/0	1	1	1	0	0
**IT-Adult**		0/2	2/2	33	26	250	4.1	1.0	8.2	0/0/1/0/0	2	1	1	0	1
**All IA**		14/14	25/28	19	90	235	4.2	1.0	8.3	1/10/11/3/2	2	2	2	1	0
**IA-Pediatric**		10/4	11/14	6	94	249	4.2	1.0	8.2	0/6/4/1/2	2	2	2	1	0
**IA-Adult**		4/10	14/14	35	86	214	4.2	1.0	8.4	1/4/7/2/0	2	2	2	1	0
***p-values, IA vs IT**		**>.99**	**>.99**	**0.86**	**0.0003**	**0.74**	**0.06**	**0.87**	**0.35**	**0.15**	**0.0071**	**0.02**	**0.03**	**0.003**	**0.86**

Supplementary Table S1. Characteristics of CHB Participants. *comparison between IA and IT Mann Whitney U for continuous variables and by Fishers Exact for categorical variables. **NA** (not available)**. IS:** insufficient sample size for statistical comparison

Supplementary Table S2.

Marker	Cell type	Isotope	Vendor	SKU#	Clone
**A. Hepatic Structural Markers (7)**					
Nuclei	Nuclear DNA	191/193 Ir	Fluidigm	201192B	
HepPar1	Hepatocytes	175Lu	Santa Cruz	sc-58693	OCH1E5
CK19	Biliary epithelial cells	150Nd	Biolegend	628502	A53-B/A2
CD31	Endothelial cells	143Nd	LSBio	LS-B15507	clone C31.3 , 7, 10
Collagen type I	Collagen	169Tm	Fluidigm	3169023D	Polyclonal
pan-Keratin	Hepatocytes and biliary epith	148Nd	Fluidigm	3148020D	C11
E-cadherin	Hepatocytes and biliary epith	158Gd	Fluidigm	3158029D	24E10
**B. HBV Markers (2)**					
HBcAg	HBV core	176Yb	Abcam	ab115992	Polyclonal
HBsAg	HBV envelope	147Sm	Biolegend	A10F1	932302
**C. Immune Markers (21)**		Isotope			
CD45	hematopoietic cells	152Sm	Fluidigm	31520118D	D9M8I
CD3	T-cells	170Er	Fluidigm	3170019D	Polyclonal
CD8	CD8 T-cells	162Dy	Fluidigm	3162035D	D8A8Y
CD4	CD4 T-cells, Mac/Mono/DC	156Gd	Fluidigm	3156033D	EPR6855
CD20	B cells	161Dy	Fluidigm	3161029D	H1
CD68	Mono/Mac/KC/DC/Granulocytes	159Tb	Fluidigm	3159035D	KP1
CD16	NK/NKT cells, inflammatory mac	146Nd	Fluidigm	3146020D	EPR16784
CD14	Mono/Mac/KC	144Nd	Fluidigm	3144025D	EPR3653
CD11b	Mono/Mac, Neutro/ NK, granulocytes	149Sm	Fluidigm	3149028D	EPR1344
CD57	NK/NKT, T and neural cells	142Nd	Fluidigm	3142007B	HCD57
HLA-ABC	broad expression (class I)	141Pr	BD	565292	EMR8-5
HLA-DR	APC's and activated cells (class II)	174Yb	Abcam	ab20181	TAL 1B5
CD45RO	Memory	173Yb	Fluidigm	3173016D	UCHL1
CD38	Activation	154Sm	Abcam	ab176886	EPR4106
CD69	Activation	166Er	Santa Cruz	sc-373798	A-5
Ki67	Proliferation	172Yb	BD	556003	B56
Granzyme B	Cytolytic effector	167Er	Fluidigm	3167021D	EPR20129-217
Perforin	Cytolytic effector	171Yb	Santa Cruz	sc-136994	F-1
PDL2	broad expression	163Dy	R&D	FAB1224A	176611
CD127 (IL7Ra)	T-cells, hepatocytes, bile ducts	168Er	Fluidigm	3168026D	EPR2955(2)
CD107a (LAMP-1)	broad expression	151Eu	FLuidigm	3151021D	H4A3

Supplementary Table S2. Panel of metal-conjugated antibodies

Supplementary Table S3.

	Groups	All	IA	IT	NC	*IA v IT v NC**	*IA vs IT***	*IA vs NC***	*IT vs NC***
		n=44	n=28	n=6	n=10	*p-values*			
**A.** Median #CD45+ cells per region of interest (ROI) acquired	**CD45+**	1,990 (1052, 2806)	2,323 (1180, 3040)	774 (529, 1283)	1,856 (1554, 2072)	*0.08*	*0.06*	*0.45*	*0.03*
	**%portal/total CD45+**	16% (8%, 28%)	26% (14%, 34%)	6% (3%, 8%)	9% (6%, 12%)	***0.003***	***0.007***	***0.016***	*0.12*
**B.** Median acquired ROI's in mm²	**Total**	1.9 (1.5,2)	1.8 (1.3, 1.9)	1.8 (1.6, 2)	2.0 (2, 2)	***0.017***	*0.64*	***0.003***	*0.30*
	**%Portal/Total ROI**	5.0% (2.5%,8.6%)	7%% (3%, 12%)	2.4% (2%, 3%)	2.9% (2%, 4%)	***0.031***	*0.07*	*0.024*	*0.59*
**C.** Median #CD45+ cells per mm² ROI (IQR25, IQR75)	**Total**	964 (768, 1424)	1,215 (806, 1646)	459 (341, 726)	933 (776, 1034)	***0.01***	***0.005***	*0.27*	***0.003***
	**Lobular**	840 (657, 1040)	958 (703, 1144)	434 (302, 685)	846 (733, 930)	***0.029***	***0.014***	*0.61*	*0.06*
	**Portal**	4,760 (1959, 5010)	4,651 (2518, 6032)	1,556 (644, 2113)	3,245 (2388, 3840)	***0.002***	***0.0027***	*0.020*	***0.011***
Lobular vs Portal	****p-value*	***<.00001***	***<.00001***	*0.06*	***0.002***				

Supplementary Table S3. Acquired regions of interests (ROI's) and their CD45+ immune cell densities in IA, IT and NC subjects. Median values for total, lobular and portal regions are shown with 25% and 75% interquartile ranges (IQR25, IQR75) from all (n=-44), immune active (IA, n=27), immune tolerant (IT, n=7) and non-infected control (NC, n=10) subjects. **A. Median number of CD45+ cells per acquired ROI per subject**; **B. Median acquired ROI's** in mm2 for ROI's per subject; **C. Median CD45+ cell density** in number of CD45+ cells per mm2 ROI. *P-values by Kruskal Wallis test (k=3) with values <0.05 considered significant; **p-values Mann Whitney U comparing various subgroups with P-values <0.0167 considered significant based on multiple comparisons. ***p-values by signed rank test comparing portal vs lobular regions within individual subjects with p-values <0.0167 considered significant.

Conflict of Interest: KBS reports research funding (Gilead), consulting (Gilead, Mirium, Up-To-Date), and DSMB (Sarepta). HLAJ reports grant support (AbbVie, Arbutus, Bristol Myers Squibb, Gilead Sciences, Janssen, Medimmune, Merck, and Roche) and consulting (Arbutus, Arena, Enyo, Gilead Sciences, GlaxoSmithKline, Janssen, Medimmune, Merck, Roche, Vir Biotechnology Inc., Viroclinics). NAT reports Institutional grant support (Gilead Sciences, GSK, and Roche-Genentech) and serves on an advisory board for Enyo Pharmaceuticals and Moderna (DSMB). MK reports institutional grant support (Gilead Sciences Inc. and Intercept Pharmaceuticals). KFM reports consulting (Gilead and Albireo). PR reports grant support (Gilead, Abbvie, Merck, Retrophin, Albireo, Mirum, and Arrowhead) and consulting (Gilead, Abbvie, Retrophin, Albireo, Mirum, Dicerna, and Audentes). SCL reports research support (Abbvie and Gilead). RKS reports grant support (Roche, Abbott, Gilead, and AbbVie), in addition to serving in DSMB for Pfizer and AskBio. DTYL reports research support (Gilead, Abbott Diagnostic, Janssen Pharmaceutical) and serving as consultant/advisor to Abbott Diagnostics. TMB reports board membership to Hepion Pharma, grant funding and stock ownership for Arbutus Biopharma, roles as co-founder and shareholder for Glycotest, and board membership for nonprofit organizations (Hepatitis B Foundation and Baruch S. Blumberg Institute and Pennsylvania Biotechnology Center). WML reports research support (Intercept, Eiger, Alexion, Novo Nordisk, Merck, BMS, Gilead, and Cumberland) and consulting activities (Genentech, Pfizer, SeaGen, Karuna, Cortexyme, and Forma Inc.). ASL reports research support from Target RWE. KMC serves on a scientific advisory board for Arbutus Inc.
